# Anion Recognition by a Pincer-Type Host Constructed from Two Polyamide Macrocyclic Frameworks Jointed by a Photo-Addressable Azobenzene Switch

**DOI:** 10.3390/ma15020692

**Published:** 2022-01-17

**Authors:** Patryk Niedbała, Magdalena Ceborska, Mart Mehmet, Wiktor Ignacak, Janusz Jurczak, Kajetan Dąbrowa

**Affiliations:** 1 Institute of Organic Chemistry, Polish Academy of Sciences, 01-224 Warsaw, Poland; pniedbala@icho.edu.pl (P.N.); mmart@icho.edu.pl (M.M.); wignacak@icho.edu.pl (W.I.); 2 Institute of Physical Chemistry, Polish Academy of Sciences, 01-224 Warsaw, Poland; mceborska@ichf.edu.pl

**Keywords:** anion recognition, macrocycle, azobenzene, pincer host, photoisomerization

## Abstract

A sterically crowded light-responsive host **1** was synthetized with a 93% yield by applying a post-functionalization protocol utilizing the double amidation of 4,4′-azodibenzoyl dichloride with a readily available 26-membered macrocyclic amine. X-ray structures of two hydrates of *trans*-**1** demonstrate a very different alignment of the azobenzene linkage, which is involved in *T*-shape or parallel-displaced π⋯π stacking interactions with the pyridine-2,6-dicarboxamide moieties from the macrocyclic backbone. Despite the rigidity of the macrocyclic framework, which generates a large steric hindrance around the azobenzene chromophore, the host **1** retains the ability to undergo a reversible *cis*⟷*trans* isomerization upon irradiation with UVA (368 nm) and blue (410 nm) light. Moreover, thermal *cis*→*trans* back-isomerization (ΔG^0^ = 106.5 kJ∙mol^−1^, *t*_½_ = 141 h) is markedly slowed down as compared to the non-macrocyclic analog. ^1^H NMR titration experiments in DMSO-*d*_6_/0.5% water solution reveal that *trans*-**1** exhibits a strong preference for dihydrogenphosphate (H_2_PO_4_^−^) over other anions (Cl^−^, MeCO_2_^−^, and PhCO_2_^−^), whereas the photogenerated metastable *cis*-**1** shows lower affinity for the H_2_PO_4_^−^ anion.

## 1. Introduction

The recognition and transport of anionic species play a central role in many fundamental chemical, biological, or environmental processes [1,2]. Numerous monographs and review papers on this subject reflect the continuing and growing interest in the supramolecular chemistry of anions [1,2]. In biological systems, anion recognition is highly selective, and it is primarily accomplished in a tailored binding cavity by multiple directional hydrogen bonds derived from amide and hydroxyl groups of amino acid residues [3,4,5,6]. Molecular recognition can be aided by additional and less-directional ionic, ion-π, hydrophobic, and stacking interactions [7,8]. Together, all of these interactions allow for the complete separation of the often initially highly solvated guest molecule from the external environment [9,10].

In artificial molecular receptors, a strong and selective binding of anions, such as chloride [11], carboxylates [12,13,14,15], and phosphates [16,17], might be realized by the selection of a proper molecular platform and decorating it with neutral (thio)amide [18,19,20], (thio)urea [19,20,21,22], pyrrole [23,24], and/or cationic ammonium [25], guanidinium [14,26], and imidazolium [27,28] binding groups. Despite enormous progress in the field of anion supramolecular chemistry in recent decades [1,2,19,29,30,31], the prediction of exact anion affinity and selectivity of a designed receptor is still a challenging task due to difficulties in simulating the correct contribution of solvation and entropy effects on the stability of the supramolecular receptor–anion complex [9,32,33,34,35,36,37]. This is particularly relevant for the more demanding chiral anion recognition, as the differences in Gibbs free energy between diastereomeric complexes are usually expected not to exceed 2 kcal∙mol^−1^.

It is therefore warranted to undertake further research focused on the design, synthesis, and evaluation of the binding properties of novel synthetic anion receptors. Such studies will ultimately lead to a better understanding of the anion complexation process and to the identification of regularities, enabling the better design of effective receptors.

**Figure 1:**
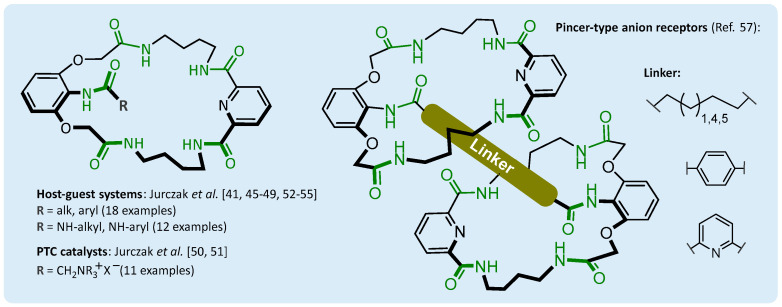
Examples of molecular structures and applications of polyamidic macrocyclic systems based on the unclosed cryptand platform; hydrogen bond donor and acceptor groups are colored in green.

Based on our work on the supramolecular recognition of anions by neutral macrocyclic tetraamides [38,39,40], we have introduced more structurally elaborate macrocyclic systems named ‘unclosed cryptands’ (UCs) [41], which contain an additional substituent (lariat arm) installed in a sterically demanding intra-annular position (Figure 1).

The lariat arm, apart from bringing extra hydrogen bond functionality, introduces a steric hindrance to the binding cavity and further pre-organizes the macrocyclic framework [41,42,43,44]. To date, this robust macrocyclic platform has been utilized for the construction of potent and selective anion receptors [41,45,46,47,48,49], phase transfer catalysts (PTC) [50,51], and closely packed molecular hosts for solid-state stabilization of transient supramolecular assemblies such as water clusters [52,53,54] and S⋯S chalcogen bonds [55]. Recently, we have developed practical and productive methods for the synthesis of these systems, using templated-macrocyclization protocols [48,55,56] and post-macrocyclization incorporation of lariat arms under mild conditions [43,46,47,56,57]. The latter strategy was utilized in the construction of a sterically crowded pincer-type system [57] acting as anion receptor for mono- and dicarboxylates. Such systems, having two macrocyclic units connected by flexible aliphatic or rigid aryl linkers, are likely not accessible using typical macrocyclization protocols due to the extensive formation of oligomeric byproducts. 

**Scheme 1:**
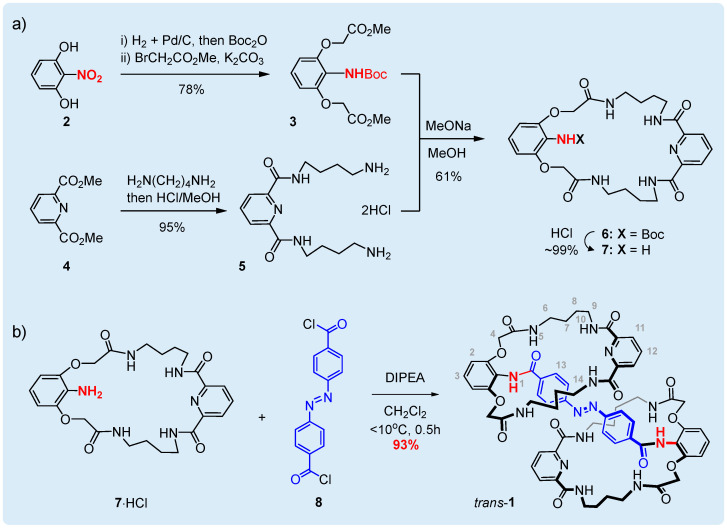
Synthesis of macrocyclic amine **4** (**a**) and sterically *C*_2_-symmetrical crowded pincer-type host *trans*-**1** by adopting an efficient post-functionalization strategy (**b**).

In the present study, we decided to expand the family of pincer-type hosts by synthesizing a novel member **1** that contains a photo-responsive linker in place of the non-dynamic linker used previously (Scheme 1) [57]. Conceptually, such structural changes enable the modification of the anion-binding properties of the host by a non-invasive and clean light stimulus [58,59,60,61,62,63].

## 2. Materials and Methods

### 2.1. Reagents and General Methods

All reagents and solvents were obtained from common suppliers and have been used as received. TLC was carried out on Merck Kieselgel F254 plates (Merck, Germany). Melting points were determined using a Boëtius M HMK hot-stage apparatus (Franz Küstner Nachf. KG, Dresden, Germany) and were uncorrected. The NMR spectra were recorded on Bruker Mercury 400 instrument (Bruker, Ettlingen, Germany) and Varian-Agilent 500 (Agilent Technologies, Inc., Santa Clara, CA, USA) instruments. Chemical shifts are reported in ppm (δ) and are set to the solvent residue peak (DMSO). *J* coupling constant values are reported in Hz. Mass spectral analyses were performed with the ESI-TOF technique on a Mariner mass spectrometer from PerSeptive Biosystem (Waltham, MA, USA). Macrocyclic amine **4** was prepared according to the previously reported procedure [56].

### 2.2. Synthethesis of Bis(N-{4,11,17,24-tetraoxo-2,26-dioxa-5,10,18,23,32-pentaazatricyclo[25.3.1.1*¹²*,*¹⁶*]dotriaconta-1(31),12(32),13,15,27,29-hexaen-31-yl}-4-[(1E)-2-[4-({4,11,17,24-tetraoxo-2,26-dioxa-5,10,18,23,32-pentaazatricyclo[25.3.1.1*¹²*,*¹⁶*]dotriaconta-1(31),12(32),13,15,27,29-hexaen-31-yl}carbamoyl)phenyl]diazen-1-yl]benzamide) (Trans-***1***)

Anhydrous HCl (~4 M solution in dioxane, 0.40 mL, ~1.6 mmol) was added dropwise at 0 °C to a solution of macrocyclic amine 4 (0.20 g, 0.33 mmol) in DCM (5 mL), resulting in the rapid precipitation of the hydrochloride salt. External cooling was removed, and the reaction mixture was stirred for 1.5 h. Then, the reaction mixture was cooled again to 0 °C and *N,N*-diisopropylethylamine (0.37 mL, 2.12 mmol) and azobenzene-4,4′-dicarbonyl dichloride (0.18 mmol) were added dropwise, and the reaction mixture was stirred for 0.5 h. Afterwards, the solvent was evaporated under vacuum, and the remaining solid residue was purified employing column chromatography and using a DCM-methanol mixture [99:1→95:5, *v/v*] as the eluent. Obtained orange oil containing *trans*-**1**∙DIPEA-HCl complex was redissolved in methanol and precipitated using water. The suspension was then sonicated for 0.5 h, filtered, and finally dried, yielding the target product *trans-***1** (0.19 g, 0.15 mmol, 93%) in the form of an orange solid.

For the hydrogen bond atom labeling, see Scheme 1. ^1^H NMR (500 MHz, DMSO-*d_6_*) δ 10.24 (s, 2H, label 1), 9.38 (t, *J* = 6.0 Hz, 4H, label 10), 8.23–8.10 (m, 14H, labels 5, 11–12, 14), 7.33 (d, *J* = 8.3 Hz, 4H, label 14), 7.27 (t, *J* = 8.4 Hz, 4H, label 3), 6.76 (d, *J* = 8.6 Hz, 4H, label 2), 4.61 (s, 8H, label 4), 3.26–3.13 (m, 16H, labels 6, 9), 1.47 (bs, 16H, labels 7–8). ^13^C NMR (126 MHz, DMSO-*d_6_*) δ 167.4, 165.3, 162.8, 153.0, 152.8, 148.9, 139.2, 136.2, 129.3, 127.9, 124.1, 121.9, 114.6, 105.3, 66.9, 38.8, 37.9, 26.3, 26.2. HRMS ESI *(m/z)* calc. for C_64_H_69_N_14_O_14_ [M–H]^−^: 1257.5118, found: 1257.5120.

### 2.3. Preparation of Host Cis-***1***

Metastable *cis*-**1** was prepared as a *trans*/*cis* mixture (containing 10–11% *cis*-**1** as deduced by the ^1^H NMR integrals) by irradiating a *trans*-**1** solution in DMSO (*c* = 5 μM, UV-Vis experiments) or DMSO-*d*_6_ (*c* = ~10 mM, ^1^H NMR experiments) with a UVA-light (Hg lamp, λ_max_ = 365 nm) for 2–5 min or 30 min, respectively. ^1^H NMR (400 MHz, DMSO-*d_6_*) δ 9.96 (s, 2H, label 1), 7.97 (m, 4H, label 10), 7.94 (m, 8H, label 11), 7.82 (m, 4H, labels 5, 12), 7.61 (t, *J* = 6.8 Hz, 4H, label 3), 7.50 (m, 8H, label 2), 6.70 (d, *J* = 8.8 Hz, 4H, label 13), 6.33 (d, *J* = 8.6 Hz, 4H, label 14), 4.55 (s, 8H, label 4), 3.07–3.05 (bd, 16H, labels 6, 9), 1.23 (bs, 16H, labels 7–8).

### 2.4. Computational Calculations

The lowest energy conformations of free hosts *trans*-**1** and *cis*-**1** and their complexes with H_2_PO_4_^−^ were found after conducting a conformational search analysis using the Spartan’20 Parallel [64] suite as previously described [58,62]. The selected conformers with the lowest energies were then optimized without any constraints at the DFT/B3LYP-D3/6-31G(d)/C-PCM:DMSO level of theory using the Spartan’20 Parallel Suite program [64].

### 2.5. Crystal Structures Measurements

Single crystals of *trans*-**1**∙(H_2_O)_2∙_MeOH suitable for X-ray crystallographic analysis were obtained by the slow diffusion of water into a solution of *trans*-**1** in methanol. The X-ray measurement of the selected monocrystal was carried out at 100(2) K on a Bruker D8 Venture Photon100 diffractometer equipped with a TRIUMPH monochromator and a MoKα fine focus sealed tube (*λ* = 0.71073 Å). A total of 1740 frames were collected with the Bruker APEX2 program [65], and the frames were integrated with the Bruker SAINT [66] software package using a narrow-frame algorithm. Data were corrected for absorption effects using the multi-scan method (SADABS) [67], and the structure was solved and refined using the SHELXTL [68] Software Package. Crystal data for *trans*-**1**∙(H_2_O)_2_∙MeOH: C_65.05_H_78.22_N_14_O_18.66_, *Mr* = 1354.84, orange prism, 0.26 × 0.19 × 0.06 mm^3^, monoclinic, space group *P*2_1_/*n*, *a* = 11.9554(8) Å, *b* = 10.5001(7) Å, *c* = 26.6855(17) Å, *β* = 91.1152(17)°, *V* = 3349.3(4) Å^3^, *Z* = 2, *D*c = 1.343 g/cm^3^, *μ*(MoKα) = 0.71073 Å, 55282 reflections measured, 5919 unique, 485 parameters, *R* = 0.0436, *wR* = 0.1013 (*R* = 0.0622, *wR* = 0.1129, all data). *GooF* = 1.020. CCDC 2128868.

Single crystals of *trans*-**1**∙(H_2_O)_3_ suitable for X-ray crystallographic analysis were obtained by a slow diffusion of water into a solution of *trans*-**1** and TBAH_2_PO_4_ in DMSO-*d*_6_/H_2_O obtained during the binding studies. The X-ray measurement of the selected monocrystal was carried out at 100 K on a SuperNova Agilent diffractometer using CuKα (λ = 1.54184 Å) radiation. Data reduction was performed with CrysAlisPro (Agilent Technologies, Version 1.171.35.21b). The structures were solved by SUPERFLIP [69] and refined using the SHELXL [68] Software Package. Crystal data for *trans*-**1**∙(H_2_O)_3_: C_64_H_70_N_14_O_20_, *Mr* = 1355.34, orange plate, 0.2 × 0.1 × 0.02 mm^3^, triclinic, space group *P*-1, *a* = 10.9112(5), *b* = 13.2394(6), *c* = 14.1760(6) Å; *α* = 91.363(4)°, *β* = 07.882(4)^o^, *γ* = 102.458(4)°, *V* = 1894.4(1) Å^3^, *Z* = 1, *D*c = 1.188 g/cm^3^, *μ*(CuK*α*) = 0.755 Å, 12375 reflections measured, 7075 unique, 472 parameters, *R* = 0.074, *wR* = 0.209 (*R* = 0.1, *wR* = 0.225, all data). *GooF* = 1.089. CCDC 2121303.

The supplementary crystallographic data for this article (CCDC 2102842 and 2102846) were deposited in the Cambridge Crystallographic Data Centre at www.ccdc.cam.ac.uk/data_request/cif (accessed on 18 December 2021) and can be obtained free of charge, by e-mailing data_request@ccdc.cam.ac.uk, or by contacting the Cambridge Crystallographic Data Centre, 12 Union Road, Cambridge CB2 1EZ, UK (fax: +44(0)1223-336033). For crystal data and structure refinement details, see Supplementary Materials.

### 2.6. Titration Experiments

Commercially available tetrabutylammonium salts (TBACl, TBAMeCO_2_, TBAPhCO_2_, and TBAH_2_PO_4_) were used as received. HPLC-.grade water was added to the commercially available DMSO-*d_6_* of 99.9% isotopic purity to obtain the appropriate solvent mixture (DMSO-*d*_6_-H_2_O 99.5:0.5% *v/v*). The host solution was titrated in an NMR tube with the solution of the respective TBA salt in receptor aliquots (details are given in Supplementary Materials). The binding constants were calculated from the changes in the chemical shifts of the amide, aliphatic, and aryl CH protons of the receptor, and from the shifts of the salt protons (aliphatic groups of the TBA cation, and the CH aryls and methyl group of benzoate and acetate anions, respectively). Non-linear curve fitting of the experimental data was carried out with the HypNMR 2008 [70,71,72] (Version 4.0.71) program using a global binding model approach [73,74]. For the determination of binding constants for *cis*-**1**, the procedure was as follows: firstly, the binding constants for the pure *trans*-**1** were determined in a separate experiment. The experiment was then repeated using a photogenerated *trans*/*cis* mixture, and fixed values for *trans*-**1** were used to fit the experimental data, allowing for the determination of the binding constants for *cis*-**1**. Using unconstrained data for *trans*-**1** produced similar results, supporting the validity of the used approach. The exact *cis*-**1** concentration was determined using the ^1^H NMR integrals and was found to be constant over the course of the experiment. Two binding models for *cis*-**1** were tested, i.e., 1:1 and mixed 1:1 + 1:2 (host:guest). Since both models predict a similar *K*_a,1_ value for *cis*-**1** and comparable binding isotherms, the simpler binding model was considered.

## 3. Results and Discussion

On the basis of our previous work on acyclic [60,61,62,75] and macrocyclic [58] photoswitchable hosts for ionic guests, we chose to use the symmetrically substituted azobenzene (AB) scaffold as a robust and reversible dynamic linker [63,76,77,78]. Preliminary considerations suggest the selection of a *para*-substituted azobenzene linker due to the possible large steric restrictions of the designed system having *ortho^−^* and *meta*-substituted linkers.

A synthesis approach for the engineered host **1** is shown in Scheme 1.

The required dihydrochloride salt of 26-membered macrocyclic amine **7** was prepared, as shown in Scheme 1a, in an overall 45% yield by following the reported protocol and using commercially available 2-nitroresorcinol **2** and dimethyl 2,6-pyridinedicarboxylate **3** as starting materials [56]. Briefly, one nitro group of **2** was catalytically reduced over 10% Pd/C in MeOH to 2-aminoresorcinol, which was reacted with Boc_2_O in a water–acetone mixture. The subsequent double O-alkylation delivered the α,ω-diester **3** with an overall yield of 78%. Dihydrochloride salt **7** was obtained by amidation of **4** in neat 1,4-diaminobutane, followed by the acidification of the so-obtained diamine with hydrochloric acid in methanol. Compounds **3** and **5** were submitted to chloride-templated macrocyclization [48,56,79,80,81,82] to produce N-Boc-protected macrocyclic compound **6** with good yield (61%), which, upon treatment with methanolic HCl, gave intermediate diamine **7**.

The target compound **1** was synthetized with a very good 93% yield as a thermodynamically stable *trans* isomer by reaction between commercially available 4,4′-azodibenzoyl dichloride **8** and two equivalents of diamine **7** in refluxing MeCN in the presence of triethylamine (Scheme 1b). The yield for the host **1** is considerably higher as compared to double amidation using shorter aliphatic and aryl linkers (55–68%) [57], indicating the expected lower steric repulsion during the synthesis of **1**.

**Figure 2:**
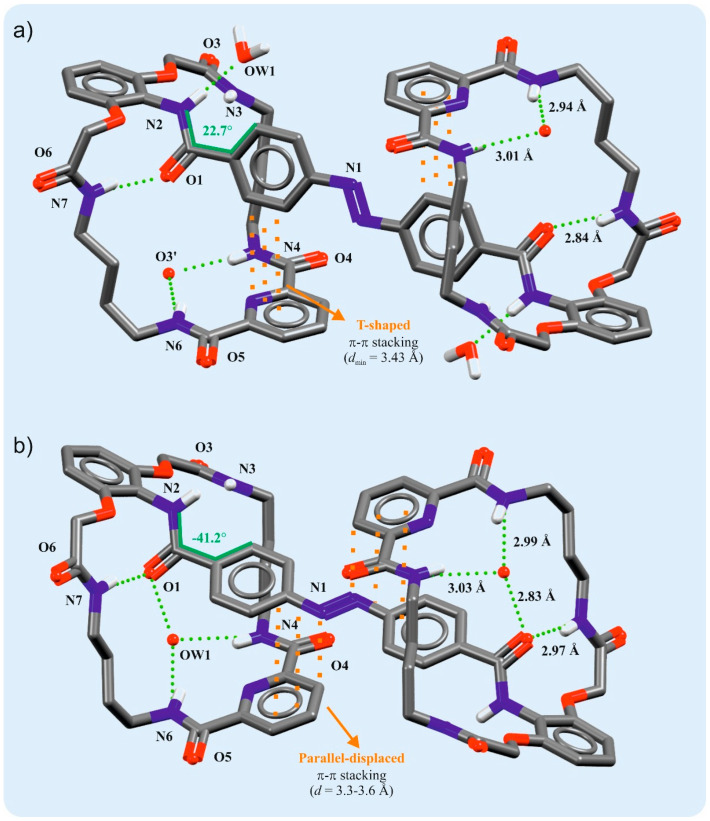
The X-ray crystal structures of solvate s of *trans*-**1,** exemplifying *T*-shape (**a**, solvate a) or parallel-displaced (**b**, solvate b) π-π stacking interactions between azobenzene and pyridine-2,6-dicarboxamide moieties; non-acidic protons, non-relevant solvent molecules and disorder have been omitted for clarity.

The monocrystals of **1** suitable for X-ray diffraction studies were obtained by a slow diffusion of water into a solution of *trans*-**1** in methanol (Figure 2a, solvate a) or into a solution of *trans*-**1** and TBAH_2_PO_4_ in DMSO-*d*_6_/H_2_O (Figure 2b, solvate b).

An analysis of the crystal structures of both solvates a and b reveals a different alignment of the linker with the *T*-shape (solvate a, *d*_π⋯π,min_ = 3.43 Å) or parallel-displaced (solvate b, *d*_π⋯π_ = 3.3–3.6 Å) π⋯π stacking interactions between AB and pyridine-2,6-dicarboxamide moieties of the same molecule. In both solvates, the AB unit is planar, whereas the adjacent carboxamide groups are twisted by 22.7° (solvate a) and −41.2° (solvate b) from the azobenzene plane. The reduced π-conjugation of the NHCO-AB moiety in solvate b is reflected by the shorter N=N bond (*d* = 1.245 vs. 1.247 Å for solvate b and a, respectively).

Further examination of the X-ray structures shows the preorganization of eight macrocyclic amide protons facing inward to the rigid macrocyclic cavity, while lariat amide protons face outward. The conformation of the macrocyclic backbone in both crystal structures is stabilized by intramolecular hydrogen bonding C(O1)⋯H-N(2) (d_O1⋯N2_ = 2.84 and 2.97 Å for solvates a and b, respectively). The host **1** in solvate a exhibits hydrogen bonding of two amide NH protons of the pyridine-2,6-dicarboxamide moiety to the carbonyl C=O(3′) group of the neighboring macrocycle (*d*_N4/N6⋯ O3′_ = 2.94, 3.01 Å), while in solvate b, the 2,6-dicarboxamide protons bind the water molecule OW1 (*d*_ON4/6**⋯OW1**_ = 2.99, 3.03 Å), which additionally interacts with the carbonyl C=O(1) group (*d*_O1**⋯OW1**_ = 2.83 Å).

Despite these differences, the conformation of the macrocyclic backbone in both solvates is comparable, indicating that the steric requirements of the rigid macrocycle scaffold have a major role in the packing of such molecules in the solid state.

**Scheme 2:**
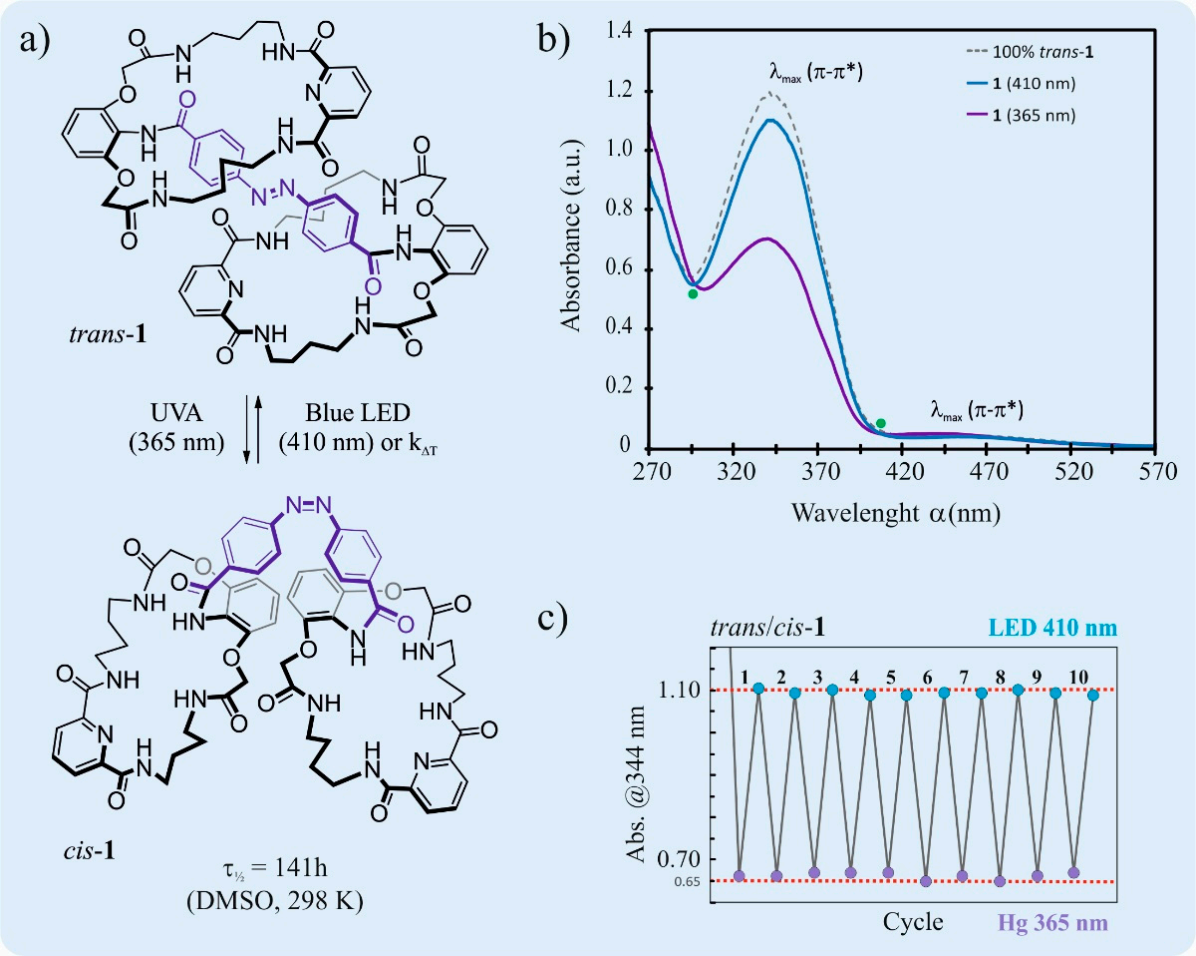
Photoswitching of host **1** in DMSO (50 μM) at 298.0 ± 0.1 K: schematic representation (**a**), UV-Vis spectra of pure *trans*-**1** and photoirradiated *trans*/*cis* mixtures (**b**), and changes of the absorbance at 344 nm upon alternate irradiation with UVA (Hg lamp 365 nm) and blue light (LED 410 nm); isobestic points in UV-Vis spectra are marked as green circles (**c**).

In the next step, the photoswitching properties of the host **1** in DMSO were investigated (Scheme 2). The metastable host *cis*-**1** was obtained as a *cis*-enriched mixture by the irradiation of a DMSO solution of *trans*-**1** (*c* = 50 μM) with a UVA light (Hg lamp, λ_max_ = 368 nm).

**Scheme 3:**
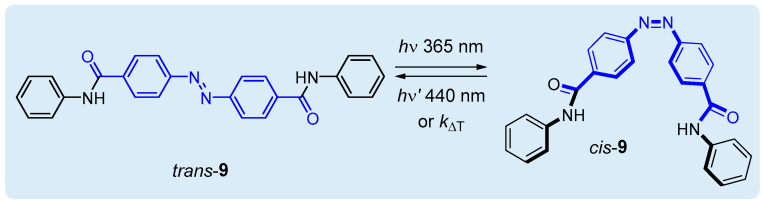
Molecular structure and photoswitching of structurally related acyclic compound **9** [83].

^1^H NMR experiments using photoirradiated samples of the host **1** (*c* = 5–10 mM) in a pure DMSO-*d*_6_ or in a DMSO-*d*_6_-H_2_O (99.5:0.5 *v/v*) solvent mixture reveal that, in a photostationary state, the content of the *cis*-isomer is relatively low (10–12%). Shinkai, Manabe, and co-workers [83] reported that the UV-photoirradiation of structurally related acyclic 4,4′-bis-N-phenylcarbamoylazobenzene **9** in a *o*-dichlorobenzene-DMF (91:9 *v/v*) solvent mixture yields only 28% of the *cis* isomer (Scheme 3).

In addition to a substantial steric hindrance of the host **1**, this may suggest extensive overlapping absorption bands for the *trans* and *cis* isomers for this type of azobenzene photosensitizer. Nevertheless, *trans* and *cis* isomers of **1** exhibit distinct spectral properties, and two isosbestic points in the UV-Vis spectra indicate that only one process is present during both photo (*trans*→*cis* and *cis*→*trans*) and thermal *cis*→*trans* isomerization (Scheme 2b). In addition, after several cycles of alternate irradiation with UVA and visible light (Blue LED, λ_max_ = 410 nm), the host **1** exhibits very good recoverability without any signs of photodegradation products (Scheme 2c). The activation entropy and enthalpy associated with the thermal *cis*→*trans* isomerization have been determined using the Eyring equation. The host **1** demonstrates positive enthalpy of activation (ΔH^‡^ = 104.8 kJ∙mol^−1^) and negative entropy of activation (ΔS^‡^ = −5.7 J∙mol^−1^∙K^−1^). Since TΔS^‡^ is relatively small (−1.7 kJ∙mol^−1^ at 298K), the Gibbs free energy of activation ΔG^‡^ is particularly high (ΔG^‡^ = 106.5 kJ∙mol^−1^), and so the host **1** demonstrates slow thermal *cis*→*trans* isomerization (*k* = 2.86∙10^−6^ s^−1^ and τ_½_ = 141 h at 298K). For comparison, acyclic compound **9** exhibits a much faster thermal decay (see Scheme *k* = 1.58∙10^−5^ s^−1^ vs. 2.85∙10^−6^ s^−1^ for compounds **9** and **1**, respectively) [83]. This supports the assumption that substantial rotational restrictions, forced by the macrocyclic ring strain, increase the steric hindrance around the azobenzene moiety, raising the thermal activation energy of the back-isomerisation.

The solution-binding properties of *trans*-**1** were determined by ^1^H NMR titration experiments in a DMSO-d6-water (99.5:0.5 *v/v*) solvent mixture. Non-coordinating and bulky tetrabutylammonium (TBA) salts of spherical chloride (Cl^−^, *p*Ka < 1), Y-shaped carboxylates: acetate (MeCO_2_^−^, *p*Ka = 4.76) and benzoate (PhCO_2_^−^, *p*Ka = 4.20), and tetrahedral dihydrogen phosphate (H_2_PO_4_^−^, *p*Ka = 2.14), acting as a dual hydrogen bond donor and acceptor, were selected as model anions featuring diverse basicities and shapes. The addition of anion aliquots caused downfield shifts of the all-amide NH resonances (i.e., 1, 5, and 10; see Scheme 1 for labels) and mixed up or downfield shifts of aryl CH resonances. Downfield shifts of NH protons are indicative of hydrogen bonding interactions with bound anions, while the mixed behavior of CH aryl protons is the result of multiple effects, such as electron density and/or conformational changes [41,60,84].

The corresponding binding isotherms, except chloride and acetate, were fitted using a mixed 1:1 and 1:2 [host:guest] binding model [85,86] and calculated association constants (*K*_a_’s) are listed in Table 1.

The data presented in Table 1 show that the host *trans*-**1** binds H_2_PO_4_^−^ with a very high affinity and a remarkable selectivity as compared to other tested anions, including a smaller and much more basic MeCO_2_^−^ (*a* = 216; see footnote of Table 1 for definition).

The origin of high selectivity for H_2_PO_4_^−^ is, among other factors, related to the dual hydrogen bond donor and acceptor properties of this anion [17]. Previous studies on UC-type receptors suggest that internal hydrogen bonding between amide groups is preserved in a solution (such as C=O(1)⋯HN(7) present in the X-ray crystal structures of *trans*-**1**; see Figure 2) [41,47,49,53,54]. Such an internal hydrogen bond has to be broken prior to the binding of Cl^−^ and carboxylates, which are hydrogen bond acceptors, to provide the proper binding conformation in which the N(2) amide proton is directed inwards toward the binding pocket. However, for binding of H_2_PO_4_^−^, this is not mandatory since this anion could also utilize P-OH groups to interact with the C=O(1) moiety as an H-bond donor, thus preserving the internal C=O(1)⋯HN(7) hydrogen bond.

It is worth emphasizing that, in biological systems, the specialized proteins employ a similar binding approach to binding phosphates with high selectivity [5,16,87]. However, in the development of the synthetic molecular receptors for H_2_PO_4_^−^, this approach has been rarely employed [88,89,90,91,92,93,94].

Receptor *trans*-**1** shows a very low affinity for chloride (*K*_a_ < 10 M^−1^), which is consistent with previous studies showing that spherical chloride does not match the 26-membered macrocyclic ring [53,57]. On the other hand, hosts possessing a smaller, 24-member macrocyclic ring show a very high affinity and selectivity for chloride [41,48,49].

Furthermore, as previously observed [57], *trans*-**1** displays a higher binding affinity for PhCO_2_^−^ over the smaller and more basic MeCO_2_^−^ (*K*_a,1_(PhCO_2_^−^)/*K*_a,1_(MeCO_2_^−^) = 7.6). It is likely that this uncommon anti-Hofmeister-type selectivity [95,96] results from the favorable π⋯π interactions between the aromatic subunits of the host **1** and the benzoate [47,97,98,99,100,101,102,103]. The literature suggests that this effect occurs for receptors containing a hydrophobic pocket in their structures [47,97,98,99,100,101,102].

The anion-binding properties of host *cis*-**1** were studied in detail only for H_2_PO_4_^−^ since other tested anions induce marginal shifts of the amide NH and aryl CH resonances, indicating very weak binding. Upon the addition of TBAH_2_PO_4_ to the *trans*/*cis* **1** mixture solution, the signals corresponding to the *cis* isomer protons shifted slightly upfield, suggesting that steric crowding is significant during the binding process (Figure S10). This in turn reduces the anion-binding affinity of a curved *cis*-**1** and deactivates the possible cooperative effect of two adjacent macrocyclic cavities. Likewise, the binding affinity of *cis*-**1** toward H_2_PO_4_^−^ (*K*_a_ = 347 M^−1^) was found to be substantially lower than for *trans*-**1**.

**Figure 3:**
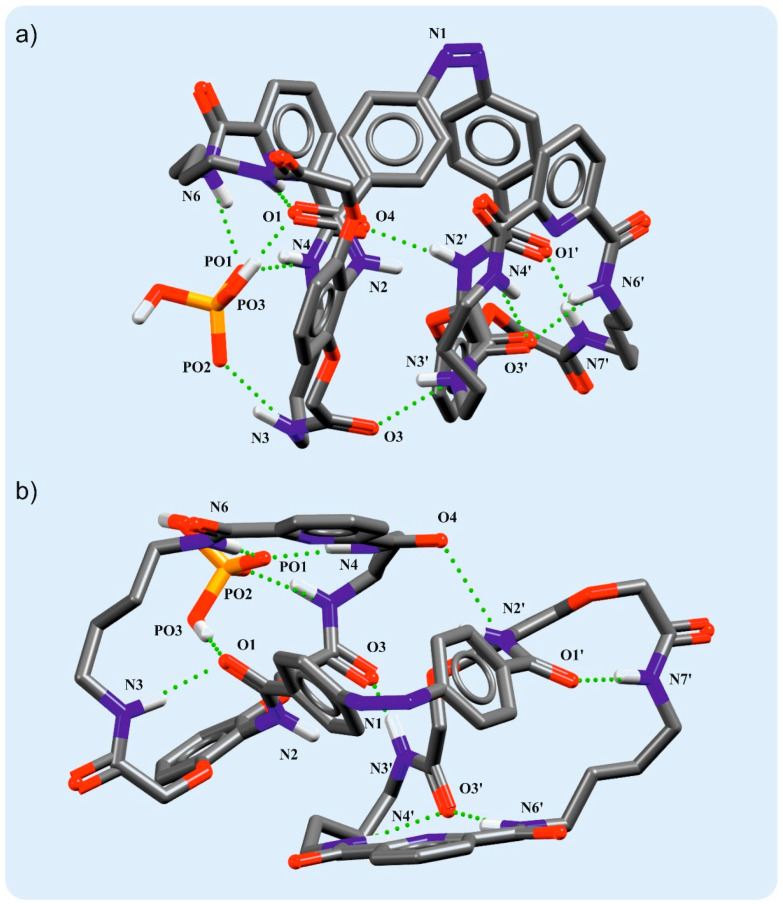
Side (**a**) and top (**b**) views of the energy-minimized structure of *cis*-**1**-H_2_PO_4_^−^ anionic complex (DFT/B3LYP-D3/6-31G(d)/C-PCM:DMSO), illustrating a highly compact host–guest conformation that is stabilized by many intramolecular hydrogen bonds; non-acidic protons have been omitted for clarity.

To obtain better insights into the reduced binding affinity of *cis*-**1** toward H_2_PO_4_^−^ in solution, we performed DFT calculations using B3LYP-D3 combined with a 6-31G(d) basis set and C-PCM model (DMSO, ε = 46.83) to approximate the solvent effects. The energy-minimized conformation of the complex of *cis*-**1** with H_2_PO_4_^−^ is demonstrated in Figure 3.

The results of DFT calculations indicate compactness of the anion complex structure. The H_2_PO_4_^−^ guest is engaged in four hydrogen bond interactions with the host *cis*-**1**. The conformation of the anionic complex is further stabilized by numerous intramolecular hydrogen bonding interactions that hinder the binding of the second guest. These results indicate that intramolecular hydrogen bonding and increased crowding cause the host **1** to be unable to adopt an optimal conformation to bind the H_2_PO_4_^−^, resulting in the decreased stability of the complex.

## 4. Conclusions

In conclusion, a new member of sterically crowded, pincer-type polyamide anion hosts containing a photoswitchable 4,4′-*di*-substituted azobenzene linker was prepared in high yield by adopting the mild and straightforward post-functionalization protocol. The crystal structure analysis of *trans*-**1** reveals that, apart from the large steric hindrance in the system, the azobenzene linkage is involved in *T*-shape or parallel-displaced π⋯π stacking interactions with the pyridine-2,6-dicarboxamide moieties from the macrocyclic backbone. The rigidity of the macrocyclic framework does not hamper the ability of the host **1** to undergo a reversible *cis*⟷*trans* isomerization upon irradiation with UVA (368 nm) and blue (410 nm) light, while it slows down the thermal *cis*→*trans* back-isomerization (ΔG^0^ = 106.5 kJ∙mol^−1^, *t*_½_ = 141 h). ^1^H NMR titration experiments in DMSO-*d*_6_/0.5% water solution reveal that *trans*-**1** exhibits a remarkable preference for dihydrogenphosphate (H_2_PO_4_^−^) over other anions (Cl^−^, MeCO_2_^−^, and PhCO_2_^−^), whereas the photogenerated metastable *cis*-**1** shows a decrease in binding affinity for the H_2_PO_4_^−^ anion.

## Figures and Tables

**Table 1:** Stability constants *K*_a_ (M^−1^) and selectivity factor α for host:guest complexes of receptor *trans*-**1** with anions ^[a]^.

Entry	Anion	*K*_a_ (Model H:G) (M^−1^)	*α* ^[b]^
1	Cl^−^	<10 (1:1)	>560
2	MeCO_2_^−^	26 (1:1)	216
3	PhCO_2_^−^	204 (1:1) 9 (1:2)	27
4	H_2_PO_4_^−^	5623 (1:1) 240 (1:2)	1

^[a]^ Determined using ^1^H NMR titration at 298 K and HypNMR 2008 software for nonlinear fitting of the binding data; anions added as TBA salts; estimated errors <10%. ^[b]^ *α* = *K_a,1_*(H_2_PO_4_^−^)/*K_a,1_*(anion)—selectivity for H_2_PO_4_^−^ over other anions.

## Data Availability

Not applicable.

## Supplementary Materials

The following are available online at https://www.mdpi.com/article/10.3390/ma15020692/s1, File S1: Copies of ^1^H and ^13^C NMR spectra of compound **1**, computational details, X-ray crystallographic data, and titration experiments for the host **1** with anions. Files S2 and S3: check CIF files for *trans*-**1**∙(H_2_O)_2_∙MeOH and *trans*-**1**∙(H_2_O)_2_. CCDC 2128868 and 2121303 references contain the supplementary crystallographic data for this paper. These data can be obtained free of charge via www.ccdc.cam.ac.uk/data_request/cif (accessed on 18 December 2021), by emailing data_request@ccdc.cam.ac.uk, or by contacting The Cambridge Crystallographic Data Centre, 12 Union Road, Cambridge CB2 1EZ, UK; fax: +44 1223 336033.

## References

[1] Chen L.J., Berry S.N., Wu X., Howe E.N.W., Gale P.A. (2020). Advances in Anion Receptor Chemistry. Chem.

[2] Zhao J., Yang D., Yang X.-J., Wu B. (2019). Anion coordination chemistry: From recognition to supramolecular assembly. Coord. Chem. Rev..

[3] Dutzler R., Campbell E.B., Cadene M., Chait B.T., MacKinnon R. (2002). X-ray structure of a ClC chloride channel at 3.0 Å reveals the molecular basis of anion selectivity. Nature.

[4] Hedstrom L. (2002). Serine Protease Mechanism and Specificity. Chem. Rev..

[5] Luecke H., Quiocho F.A. (1990). High specificity of a phosphate transport protein determined by hydrogen bonds. Nature.

[6] Pflugrath J.W., Quiocho F.A. (1985). Sulphate sequestered in the sulphate-binding protein of *Salmonella typhimurium* is bound solely by hydrogen bonds. Nature.

[7] Alkorta I., Elguero J., Frontera A. (2020). Not Only Hydrogen Bonds: Other Noncovalent Interactions. Crystals.

[8] Molina P., Zapata F., Caballero A. (2017). Anion Recognition Strategies Based on Combined Noncovalent Interactions. Chem. Rev..

[9] Liu Y., Parks F.C., Sheetz E.G., Chen C.-H., Flood A.H. (2021). Polarity-Tolerant Chloride Binding in Foldamer Capsules by Programmed Solvent-Exclusion. J. Am. Chem. Soc..

[10] Fernández A., Crespo A. (2008). Protein wrapping: A molecular marker for association, aggregation and drug design. Chem. Soc. Rev..

[11] Dabrowa K., Ulatowski F., Lichosyt D., Jurczak J. (2017). Catching the chloride: Searching for non-Hofmeister selectivity behavior in systematically varied polyamide macrocyclic receptors. Org. Biomol. Chem..

[12] Ulatowski F., Jurczak J. (2016). Chiral Recognition of Carboxylates—Receptors, Analytical Tools, and More. Asian J. Org. Chem..

[13] Niedbała P., Dąbrowa K., Wasiłek S., Jurczak J. (2021). Recognition of Chiral Carboxylates by Synthetic Receptors. Molecules.

[14] Blondeau P., Segura M., Perez-Fernandez R., de Mendoza J. (2007). Molecular recognition of oxoanions based on guanidinium receptors. Chem. Soc. Rev..

[15] Fitzmaurice R.J., Kyne G.M., Douheret D., Kilburn J.D. (2002). Synthetic receptors for carboxylic acids and carboxylates. J. Chem. Soc. Perkin Trans. 1.

[16] Hirsch A.K., Fischer F.R., Diederich F. (2007). Phosphate recognition in structural biology. Angew. Chem. Int. Ed. Engl..

[17] Hargrove A.E., Nieto S., Zhang T., Sessler J.L., Anslyn E.V. (2011). Artificial Receptors for the Recognition of Phosphorylated Molecules. Chem. Rev..

[18] Kang S.O., Begum R.A., Bowman-James K. (2006). Amide-based ligands for anion coordination. Angew. Chem. Int. Ed. Engl..

[19] Manna U., Das G. (2021). An overview of anion coordination by hydroxyl, amine and amide based rigid and symmetric neutral dipodal receptors. Coord. Chem. Rev..

[20] Dydio P., Lichosyt D., Jurczak J. (2011). Amide-and urea-functionalized pyrroles and benzopyrroles as synthetic, neutral anion receptors. Chem. Soc. Rev..

[21] Blažek Bregović V., Basarić N., Mlinarić-Majerski K. (2015). Anion binding with urea and thiourea derivatives. Coord. Chem. Rev..

[22] Li A.-F., Wang J.-H., Wang F., Jiang Y.-B. (2010). Anion complexation and sensing using modified urea and thiourea-based receptors. Chem. Soc. Rev..

[23] Vargas-Zúñiga G.I., Sessler J.L. (2017). Pyrrole N–H anion complexes. Coord. Chem. Rev..

[24] Sessler J.L., Camiolo S., Gale P.A. (2003). Pyrrolic and polypyrrolic anion binding agents. Coord. Chem. Rev..

[25] Llinares J.M., Powell D., Bowman-James K. (2003). Ammonium based anion receptors. Coord. Chem. Rev..

[26] Houk R.J., Tobey S.L., Anslyn E.V. (2005). Abiotic guanidinium receptors for anion molecular recognition and sensing. Anion Sens..

[27] Xu Z., Kim S.K., Yoon J. (2010). Revisit to imidazolium receptors for the recognition of anions: Highlighted research during 2006–2009. Chem. Soc. Rev..

[28] Yoon J., Kim S.K., Singh N.J., Kim K.S. (2006). Imidazolium receptors for the recognition of anions. Chem. Soc. Rev..

[29] Taylor M.S. (2020). Anion recognition based on halogen, chalcogen, pnictogen and tetrel bonding. Coord. Chem. Rev..

[30] Pancholi J., Beer P.D. (2020). Halogen bonding motifs for anion recognition. Coord. Chem. Rev..

[31] Wu X., Gilchrist A.M., Gale P.A. (2020). Prospects and Challenges in Anion Recognition and Transport. Chem.

[32] Zimnicka M., Kozłowska K., Danikiewicz W. (2020). Beyond Size Complementary Factors in Anion–Tetralactam Macrocycle Complexes: From Intrinsic Gas-Phase to Solvent-Predicted Stabilities. J. Org. Chem..

[33] Sherbow T.J., Fargher H.A., Haley M.M., Pluth M.D., Johnson D.W. (2020). Solvent-Dependent Linear Free-Energy Relationship in a Flexible Host–Guest System. J. Org. Chem..

[34] Liu Y., Sengupta A., Raghavachari K., Flood A.H. (2017). Anion binding in solution: Beyond the electrostatic regime. Chem.

[35] Sengupta A., Liu Y., Flood A.H., Raghavachari K. (2018). Anion-Binding Macrocycles Operate Beyond the Electrostatic Regime: Interaction Distances Matter. Chem. Eur. J..

[36] Cockroft S.L. (2017). Screening Solvent Effects in Anion Recognition. Chem.

[37] Jeanmairet G., Levesque M., Borgis D. (2020). Tackling Solvent Effects by Coupling Electronic and Molecular Density Functional Theory. J. Chem. Theory Comput..

[38] Chmielewski M.J., Jurczak J. (2006). Anion Binding versus Intramolecular Hydrogen Bonding in Neutral Macrocyclic Amides. Chem. Eur. J..

[39] Chmielewski M.J., Jurczak J. (2005). Anion Recognition by Neutral Macrocyclic Amides. Chem. Eur. J..

[40] Chmielewski M., Jurczak J. (2004). Size complementarity in anion recognition by neutral macrocyclic tetraamides. Tetrahedron Lett..

[41] Dąbrowa K., Pawlak M., Duszewski P., Jurczak J. (2012). “Unclosed Cryptands”: A Point of Departure for Developing Potent Neutral Anion Receptors. Org. Lett..

[42] Jurczak J., Sobczuk A., Dąbrowa K., Lindner M., Niedbała P., Stepniak P. (2018). Chirality of 20-membered unclosed cryptand: Macroring distortion via lariat arm modification. Chirality.

[43] Jurczak J., Sobczuk A., Da Browa K., Lindner M., Niedbala P. (2018). An Indirect Synthetic Approach toward Conformationally Constrained 20-Membered Unclosed Cryptands via Late-Stage Installation of Intraannular Substituents. J. Org. Chem..

[44] Ziach K., Dąbrowa K., Niedbała P., Kalisiak J., Jurczak J. (2016). Exploration of structural motifs influencing solid-state conformation and packing of unclosed cryptands sharing the same 19-membered macrocyclic core. Tetrahedron.

[45] Niedbała P., Jurczak J. (2020). Effective synthetic strategy towards highly selective macrocyclic anion receptors based on static combinatorial chemistry. Tetrahedron.

[46] Niedbała P., Jurczak J. (2020). One-Pot Parallel Synthesis of Unclosed Cryptands—Searching for Selective Anion Receptors via Static Combinatorial Chemistry Techniques. ACS Omega.

[47] Niedbała P., Majdecki M., Dąbrowa K., Jurczak J. (2020). Selective Carboxylate Recognition Using Urea-Functionalized Unclosed Cryptands: Mild Synthesis and Complexation Studies. J. Org. Chem..

[48] Dąbrowa K., Lindner M., Tyszka-Gumkowska A., Jurczak J. (2021). Imino-thiolate-templated synthesis of a chloride-selective neutral macrocyclic host with a specific “turn-off-on” fluorescence response for hypochlorite (ClO^−^). Org. Chem. Front..

[49] Dąbrowa K., Lindner M., Wasiłek S., Jurczak J. (2020). Selective Recognition of Chloride by a 24-Membered Macrocyclic Host with a Hydrophobic Methylenepyrene Substituent. Eur. J. Org. Chem..

[50] Tyszka-Gumkowska A., Jurczak J. (2020). A General Method for High-Pressure-Promoted Postfunctionalization of Unclosed Cryptands: Potential Phase-Transfer Catalysts. J. Org. Chem..

[51] Tyszka-Gumkowska A., Jurczak J. (2021). Divergent synthesis of pyrrolidine and glutamic acid derivatives using a macrocyclic phase-transfer catalyst under high-pressure conditions. Org. Chem. Front..

[52] Dąbrowa K., Ceborska M., Jurczak J. (2018). Solid-state entrapment of water clusters by 26-membered pentamide unclosed cryptands –probing the para-substituent effect. Supramol. Chem..

[53] Dąbrowa K., Ceborska M., Jurczak J. (2014). Trapping of Octameric Water Cluster by the Neutral Unclosed Cryptand Environment. Cryst. Growth Des..

[54] Dabrowa K., Ceborska M., Jurczak J. (2021). Stabilization of Near Identical Hydrogen Bonded Octameric Water Clusters in Crystal Structures of Three Distinct Non-Charged Polyamide Macrocyclic Host Molecules. Molecules.

[55] Dąbrowa K., Ceborska M., Pawlak M., Jurczak J. (2017). Comparative Structural Studies of Four Homologous Thioamidic Unclosed Crytpands: Self-Encapsulation of Lariat Arm, Odd–Even Effects, Anomalously Short S· ·S Chalcogen Bonding, and More. Cryst. Growth Des..

[56] Dabrowa K., Niedbala P., Majdecki M., Duszewski P., Jurczak J. (2015). A General Method for Synthesis of Unclosed Cryptands via H-Bond Templated Macrocyclization and Subsequent Mild Postfunctionalization. Org. Lett..

[57] Niedbała P., Jurczak J. (2021). A new class of “pincer” receptors–macrocyclic systems containing an incorporated amide group. J. Coord. Chem..

[58] Sokolowska P., Dabrowa K., Jarosz S. (2021). Visible-Light Responsive Sucrose-Containing Macrocyclic Host for Cations. Org. Lett..

[59] Tecilla P., Bonifazi D. (2020). Configurational Selection in Azobenzene-Based Supramolecular Systems Through Dual-Stimuli Processes. ChemistryOpen.

[60] Dąbrowa K., Jurczak J. (2017). Tetra-(meta-butylcarbamoyl) azobenzene: A Rationally Designed Photoswitch with Binding Affinity for Oxoanions in a Long-Lived Z-State. Org. Lett..

[61] Dąbrowa K., Niedbała P., Jurczak J. (2016). Engineering Light-Mediated Bistable Azobenzene Switches Bearing Urea d-Aminoglucose Units for Chiral Discrimination of Carboxylates. J. Org. Chem..

[62] Dabrowa K., Niedbala P., Jurczak J. (2014). Anion-tunable control of thermal Z → E isomerisation in basic azobenzene receptors. Chem. Commun..

[63] Lee S., Flood A.H. (2013). Photoresponsive receptors for binding and releasing anions. J. Phys. Org. Chem..

[64] (2020). Spartan’20 for Windows.

[65] (2013). APEX2.

[66] (2013). SAINT.

[67] (2012). SADABS.

[68] Sheldrick G. (2015). Crystal structure refinement with SHELXL. Acta Crystallogr. Sect. C.

[69] Palatinus L., Chapuis G. (2007). SUPERFLIP—A computer program for the solution of crystal structures by charge flipping in arbitrary dimensions. J. Appl. Crystallogr..

[70] Rodríguez-Barrientos D., Rojas-Hernández A., Gutiérrez A., Moya-Hernández R., Gómez-Balderas R., Ramírez-Silva M.T. (2009). Determination of pKa values of tenoxicam from 1H NMR chemical shifts and of oxicams from electrophoretic mobilities (CZE) with the aid of programs SQUAD and HYPNMR. Talanta.

[71] Frassineti C., Alderighi L., Gans P., Sabatini A., Vacca A., Ghelli S. (2003). Determination of protonation constants of some fluorinated polyamines by means of 13C NMR data processed by the new computer program HypNMR2000. Protonation sequence in polyamines. Anal. Bioanal. Chem..

[72] Frassineti C., Ghelli S., Gans P., Sabatini A., Moruzzi M.S., Vacca A. (1995). Nuclear Magnetic Resonance as a Tool for Determining Protonation Constants of Natural Polyprotic Bases in Solution. Anal. Biochem..

[73] Lowe A.J., Pfeffer F.M., Thordarson P. (2012). Determining binding constants from 1H NMR titration data using global and local methods: A case study using [n]polynorbornane-based anion hosts. Supramol. Chem..

[74] Thordarson P. (2011). Determining association constants from titration experiments in supramolecular chemistry. Chem. Soc. Rev..

[75] Ulatowski F., Dąbrowa K., Jurczak J. (2016). Supramolecular detection of geometrical differences of azobenzene carboxylates. Tetrahedron Lett..

[76] Jerca F.A., Jerca V.V., Hoogenboom R. (2022). Advances and opportunities in the exciting world of azobenzenes. Nat. Rev. Chem..

[77] Łukasik N., Chojnacki J., Luboch E., Okuniewski A., Wagner-Wysiecka E. (2020). Photoresponsive, amide-based derivative of embonic acid for anion recognition. J. Photochem. Photobiol. A.

[78] Wagner-Wysiecka E., Łukasik N., Biernat J.F., Luboch E. (2018). Azo group(s) in selected macrocyclic compounds. J. Incl. Phenom. Macrocycl. Chem..

[79] Vidović N., Horvat G., Riva D., Rinkovec T., Cindro N., Tomišić V., Speranza G. (2020). Chloride-Assisted Peptide Macrocyclization. Org. Lett..

[80] Łęczycka-Wilk K., Dabrowa K., Cmoch P., Jarosz S. (2017). Chloride-Templated Macrocyclization and Anion-Binding Properties of C^2^-Symmetric Macrocyclic Ureas from Sucrose. Org. Lett..

[81] Martí-Centelles V., Burguete M.I., Luis S.V. (2016). Macrocycle Synthesis by Chloride-Templated Amide Bond Formation. J. Org. Chem..

[82] Satake A., Ishizawa Y., Katagiri H., Kondo S.-I. (2016). Chloride Selective Macrocyclic Bisurea Derivatives with 2,2′-Binaphthalene Moieties as Spacers. J. Org. Chem..

[83] Shinkai S., Honda Y., Minami T., Ueda K., Manabe O., Tashiro M. (1983). Photoresponsive crown ethers. 9. Cylindrical and phane crown ethers with azobenzene segments as a light-switch functional group. Bull. Chem. Soc. Jpn..

[84] Amendola V., Fabbrizzi L., Mosca L. (2010). Anion recognition by hydrogen bonding: Urea-based receptors. Chem. Soc. Rev..

[85] Brynn Hibbert D., Thordarson P. (2016). The death of the Job plot, transparency, open science and online tools, uncertainty estimation methods and other developments in supramolecular chemistry data analysis. Chem. Commun..

[86] Ulatowski F., Dąbrowa K., Bałakier T., Jurczak J. (2016). Recognizing the Limited Applicability of Job Plots in Studying Host–Guest Interactions in Supramolecular Chemistry. J. Org. Chem..

[87] Wang Z., Choudhary A., Ledvina P.S., Quiocho F.A. (1994). Fine tuning the specificity of the periplasmic phosphate transport receptor. Site-directed mutagenesis, ligand binding, and crystallographic studies. J. Biol. Chem..

[88] Dąbrowa K., Niedbała P., Pawlak M., Lindner M., Ignacak W., Jurczak J. (2020). Tuning Anion-Binding Properties of 22-Membered Unclosed Cryptands by Structural Modification of the Lariat Arm. ACS Omega.

[89] Cho J., Verwilst P., Kang M., Pan J.L., Sharma A., Hong C.S., Kim J.S., Kim S. (2020). Crown ether-appended calix [2] triazolium [2] arene as a macrocyclic receptor for the recognition of the H_2_PO_4_^−^ anion. Chem. Commun..

[90] Yakimova L., Shurpik D., Stoikov I. (2016). Amide-functionalized pillar [5] arenes as a novel class of macrocyclic receptors for the sensing of H_2_PO_4_^−^ anion. Chem. Commun..

[91] Deliomeroglu M.K., Lynch V.M., Sessler J.L. (2016). Non-cyclic formylated dipyrromethanes as phosphate anion receptors. Chem. Sci..

[92] Kataev E.A., Shumilova T.A. (2015). Investigation of structural mimetics of natural phosphate ion binding motifs. Molecules.

[93] Kondo S., Takai R. (2013). Selective detection of dihydrogen phosphate anion by fluorescence change with tetraamide-based receptors bearing isoquinolyl and quinolyl moieties. Org. Lett..

[94] Mahanta S.P., Kumar B.S., Panda P.K. (2011). Meso-diacylated calix [4] pyrrole: Structural diversities and enhanced binding towards dihydrogenphosphate ion. Chem. Commun..

[95] Martin K., Nõges J., Haav K., Kadam S.A., Pung A., Leito I. (2017). Exploring Selectivity of 22 Acyclic Urea-, Carbazole- and Indolocarbazole-Based Receptors towards 11 Monocarboxylates. Eur. J. Org. Chem..

[96] Kadam S.A., Martin K., Haav K., Toom L., Mayeux C., Pung A., Gale P.A., Hiscock J.R., Brooks S.J., Kirby I.L. (2015). Towards the Discrimination of Carboxylates by Hydrogen-Bond Donor Anion Receptors. Chem. Eur. J..

[97] Chang K.-C., Chen C.-Y., Lin T.-P., Ku P.-J., Chen C.-L., Wang C.-M., Lin H.-M., Tseng M.-C., Singh A.S., Sun S.-S. (2018). Platinum (II)-directed Self-assembly Loop Complexes for Anion Recognition and Sensing. J. Chin. Chem. Soc..

[98] Hudeček O., Budka J., Dvořáková H., Cuřínová P., Císařová I., Lhoták P. (2013). Anion receptors based on ureidocalix [4] arenes immobilised in the partial cone conformation. New J. Chem..

[99] Young P.G., Jolliffe K.A. (2012). Selective recognition of sulfate ions by tripodal cyclic peptides functionalised with (thio)urea binding sites. Org. Biomol. Chem..

[100] Casnati A., Sansone F., Ungaro R. (2003). Peptido- and Glycocalixarenes:  Playing with Hydrogen Bonds around Hydrophobic Cavities. Acc. Chem. Res..

[101] Sansone F., Baldini L., Casnati A., Lazzarotto M., Ugozzoli F., Ungaro R. (2002). Biomimetic macrocyclic receptors for carboxylate anion recognition based on C-linked peptidocalix [4] arenes. Proc. Natl. Acad. Sci. USA.

[102] Lazzarotto M., Sansone F., Baldini L., Casnati A., Cozzini P., Ungaro R. (2001). Synthesis and Properties of Upper Rim C-Linked Peptidocalix [4] arenes. Eur. J. Org. Chem..

[103] Beer P.D. (1997). Solvent dependent anion selectivity exhibited by neutral ferrocenoyl receptors. Chem. Commun..

