# Supplementary material for: Anion Recognition by a Pincer-Type Host Constructed from Two Polyamide Macrocyclic Frameworks Jointed by a Photo-Addressable Azobenzene Switch
Source: Materials (Basel). 2022 Jan 17;15(2):692. doi: 10.3390/ma15020692 (PMC8777895; doi:10.3390/ma15020692)
Supplement: Supplementary file 1 [file materials-15-00692-s001.zip › materials-1539615-supplementary.pdf]

Supporting Information

Anion recognition by pincer-type host constructed from two polyamide macrocyclic frameworks jointed by photo-addressable azobenzene switch

Patryk Niedbała,[1] Magdalena Ceborska,[2] Mehmet Mart,[1] Wiktor Ignacak,[1] Janusz Jurczak,*[1] and Kajetan Dąbrowa[1]

[1] Institute of Organic Chemistry, Polish Academy of Sciences, Kasprzaka 44/52, 01-224 Warsaw, Poland.

[2] Institute of Physical Chemistry, Polish Academy of Sciences, Kasprzaka 44/52, 01-224 Warsaw, Poland.

E-mail: kdabrowa@icho.edu.pl, jurczak_group@icho.edu.pl

Contents

1. COPIES OF ^{1}H, ^{13}C, AND 2D NMR SPECTRA 2
2. TITRATION EXPERIMENTS 9
3. X-RAY MEASUREMENTS DETAILS 11
4. REFERENCES 15

1. Copies of ^{1}H, ^{13}C, and 2D NMR spectra

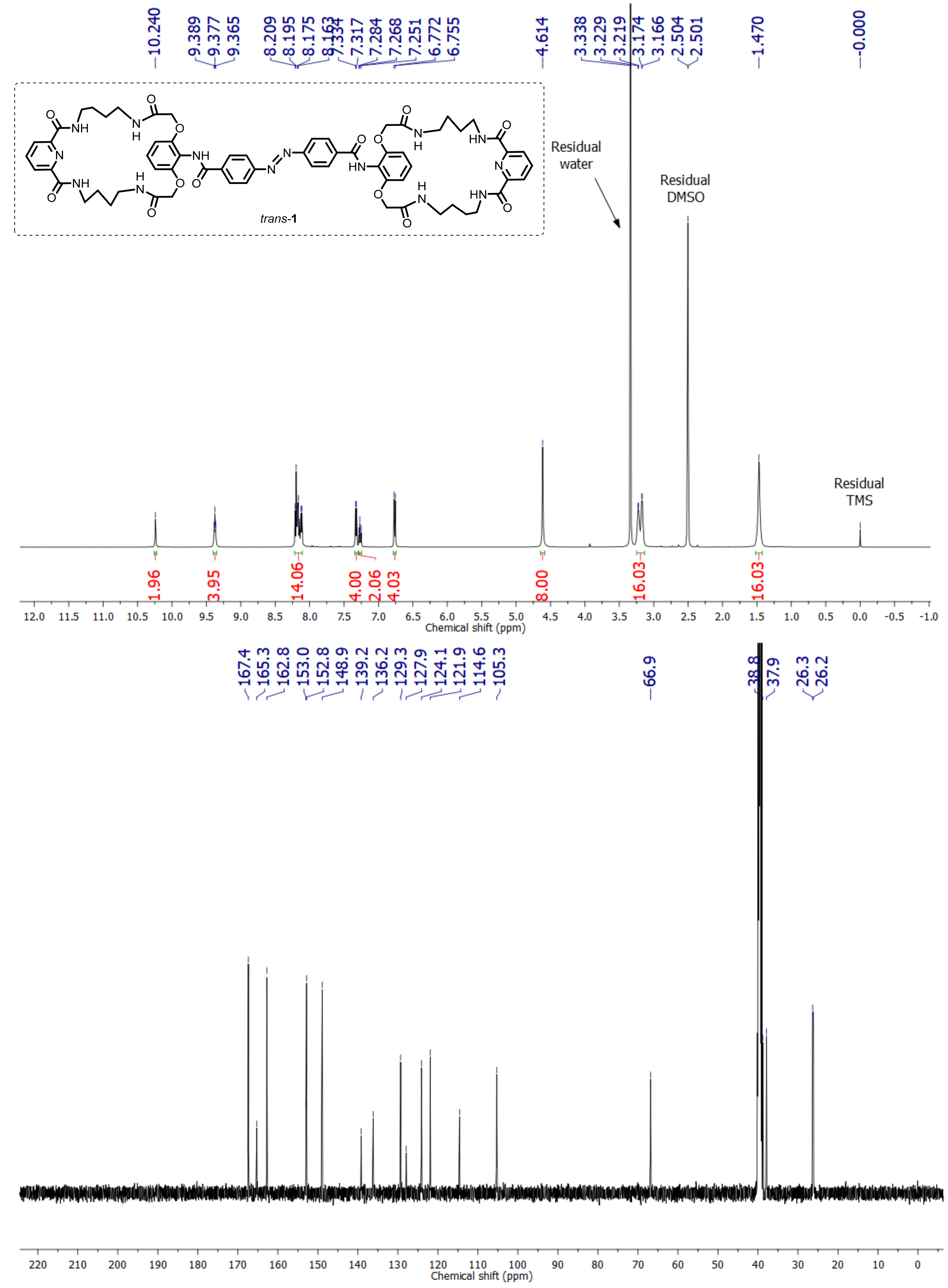

Figure S1. ^{1}H NMR (500 MHz) and $^{13}C\{^{1}H\}$ NMR (126 MHz) spectra of compound *trans*-**1** in DMSO-d_6.

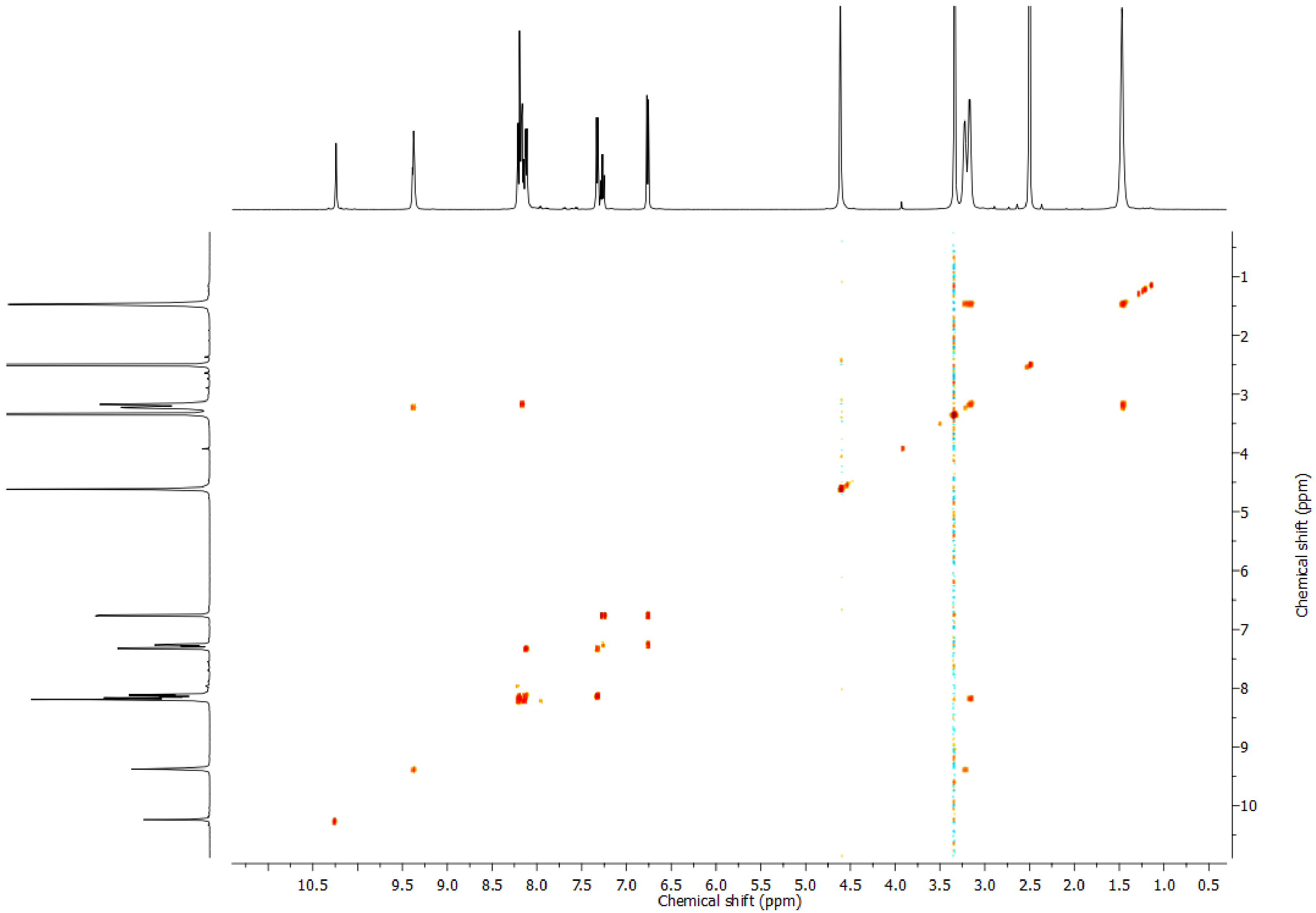

Figure S2. COSY spectrum of compound *trans*-**1** in DMSO-d_6.

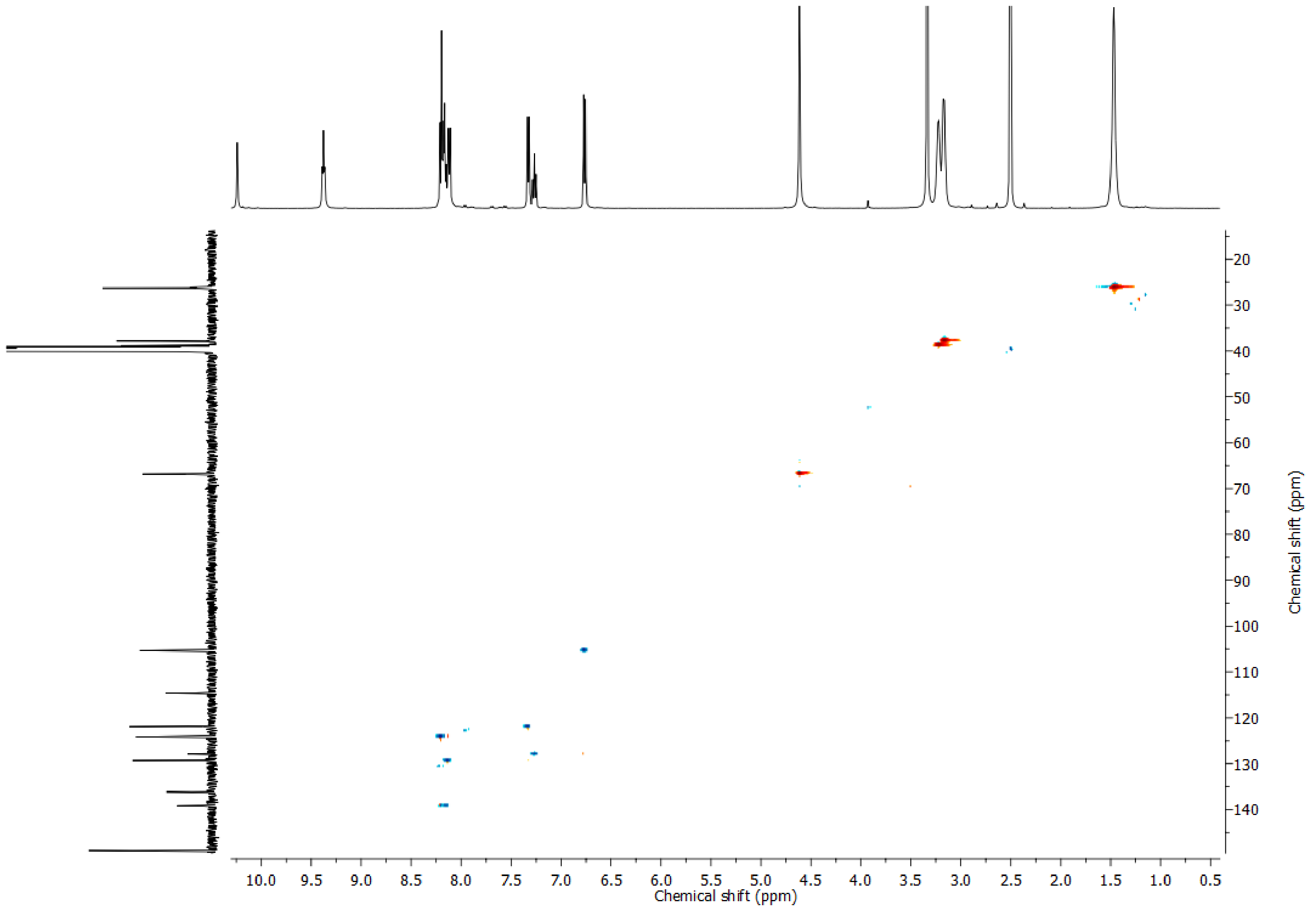

Figure S3. HSQC spectrum of compound *trans*-**1** in DMSO-d_6.

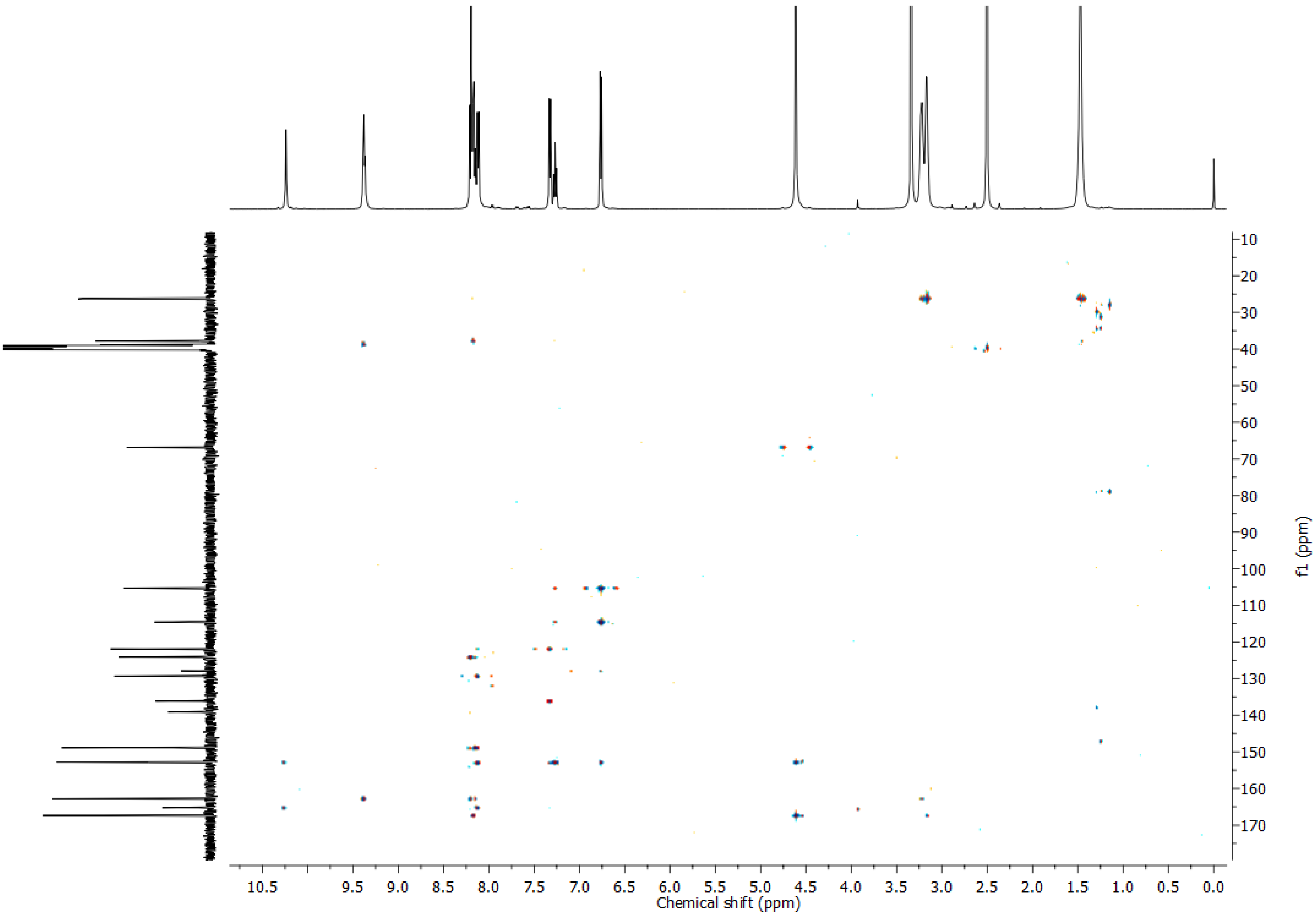

Figure S4. HMBC spectrum of compound *trans*-**1** in DMSO-d_6.

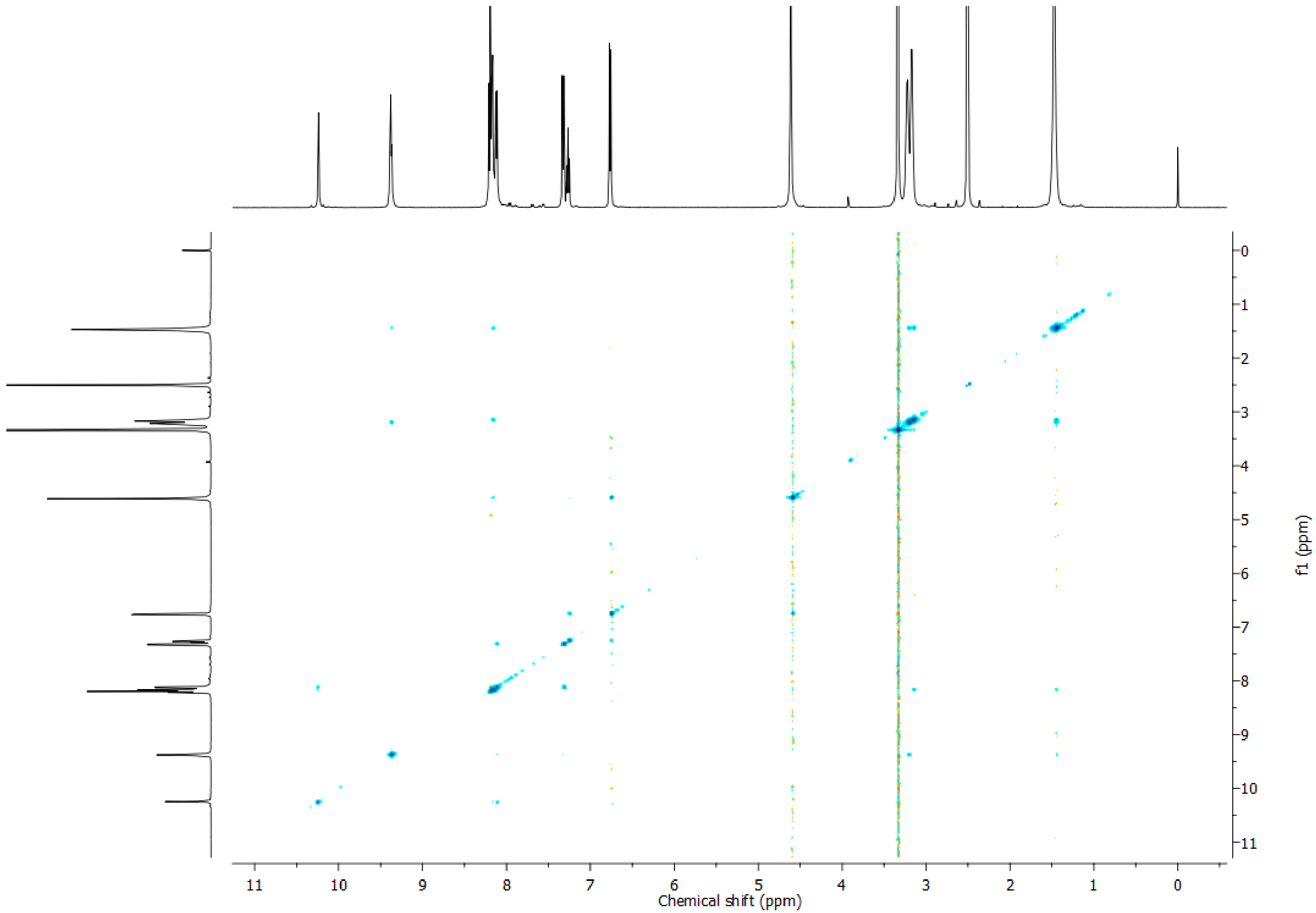

Figure S5. NOESY spectrum of compound *trans*-**1** in DMSO-d_6.

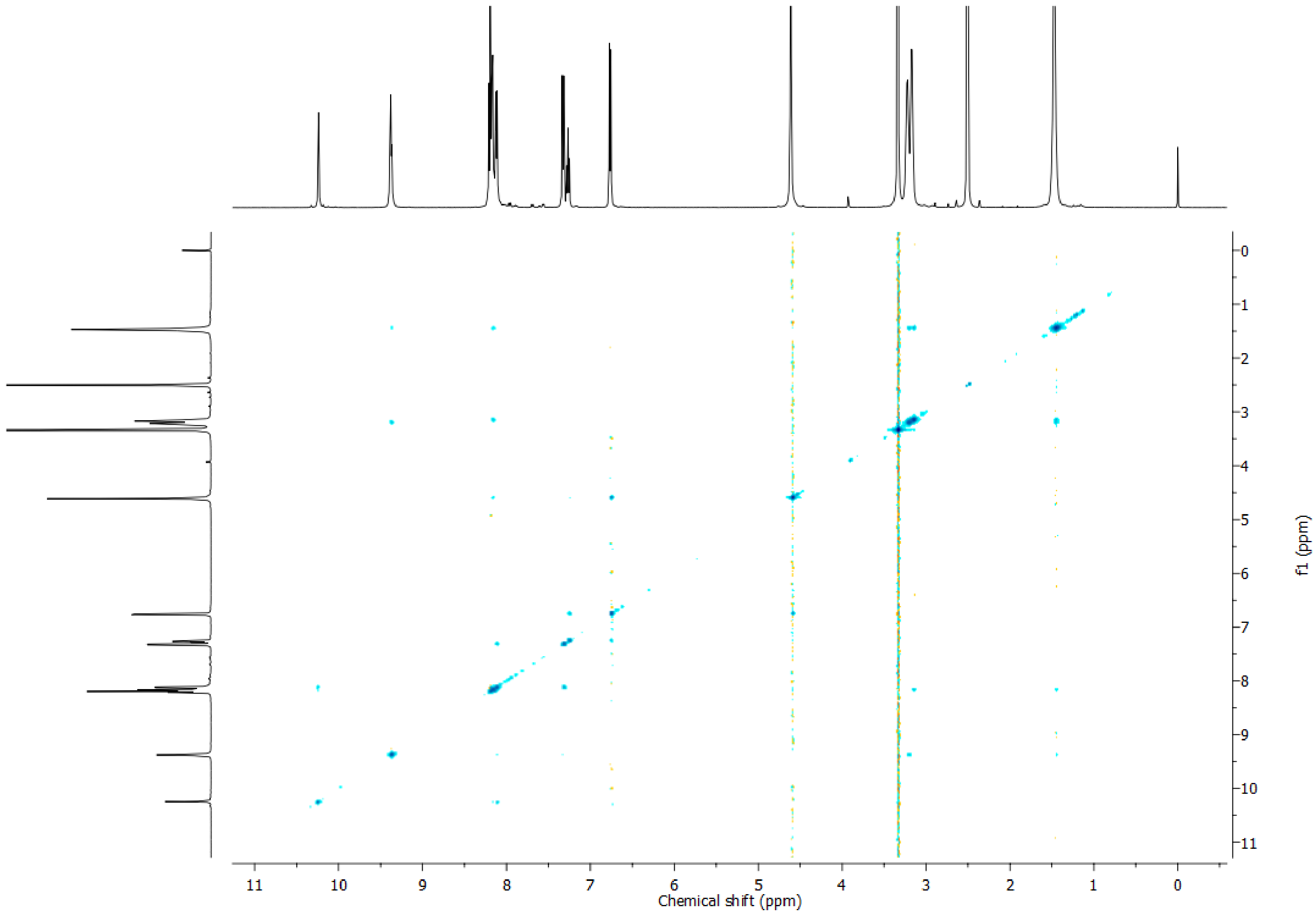

Figure S6. NOESY spectrum of compound *trans*-**1** in DMSO-d_6.

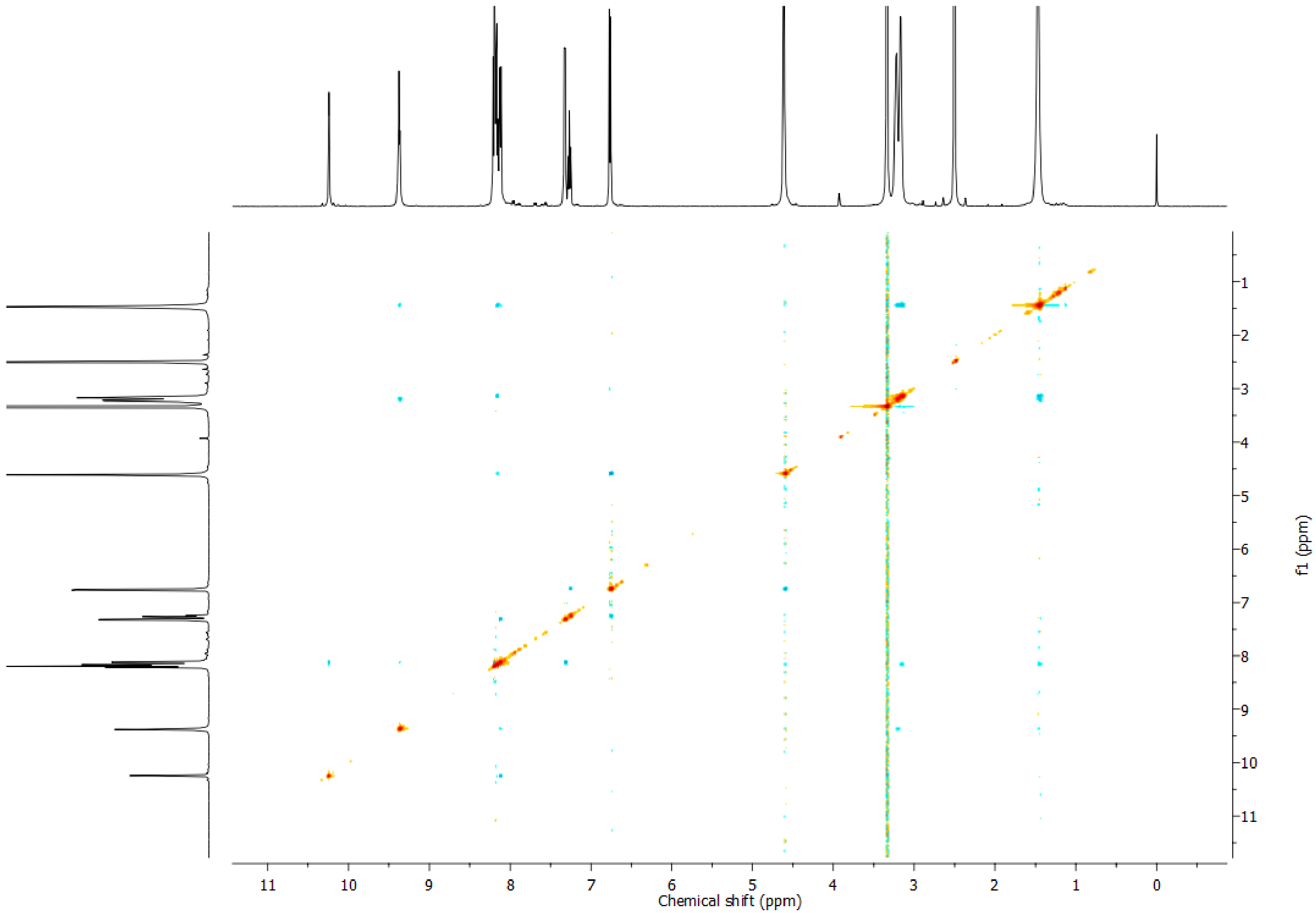

Figure S7. ROESY spectrum of compound *trans*-**1** in DMSO-d_6.

2. Titration experiments

As the source of anions, commercially available tetrabutylammonium salts were used. HPLC grade water was added to the commercially available DMSO-d_6 of 99.9% isotopic purity to obtain the appropriate water concentration. The host solution was titrated in a NMR tube with the solution of the respective TBA salt in receptor aliquots. The binding constants were calculated from the changes in chemical shifts of ligand protons which were shifted during titration. Nonlinear curve fitting was carried out with HypNMR 2008[1] (Version 4.0.71) program with fitting to the appropriate global binding model [2,3].

Table. S1. Titration details, global stability constants K_a (M^{-1}) for receptors *trans*-**1** and *cis*-**1** with various anions in DMSO-d_6 + 0.5% H_2O[a]

Entry	Receptor	Anion	C_{Host} (M)	C_{Guest} (M)	K_a (M^{-1})
1	*trans*-**1**	Cl^-	0.01002	0.74425	< 10 (1:1)
2		$MeCO_2^-$	0.01002	0.38074	26 (1:1)
3		$PhCO_2^-$	0.01002	0.36197	204 (1:1)<br>9 (1:2)
4		$H_2PO_4^-$	0.01002	0.15238	5623 (1:1)<br>240 (1:2)
5	*cis*-**1**[b]	$H_2PO_4^-$	0.00988[c]	0.15957	347 (1:1)[d]

[a] Values determined by ^1H NMR titration experiments at T = 298 K using HypNMR 2008 software,[1] errors < 10%, TBA salts as the source of anions. Experiment carried out in dark. [b] For host-1, stability constants determined in a separate experiment were used to fit the binding data (Table S1, entry 4). [c] Total concentration of a *trans*/*cis* mixture of host **1**. [d] Two binding models for *cis*-**1** were tested, i.e. 1:1 and mixed 1:1 + 1:2 (host:guest). Since both models predict a similar $K_{a,1}$ value for *cis*-**1** and comparable binding isotherms, the simpler binding model was considered.

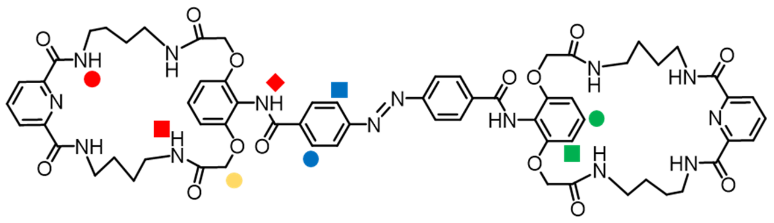

trans-**1**

Figure S8. Labeling of the selected proton peaks of the receptor *trans*-**1**.

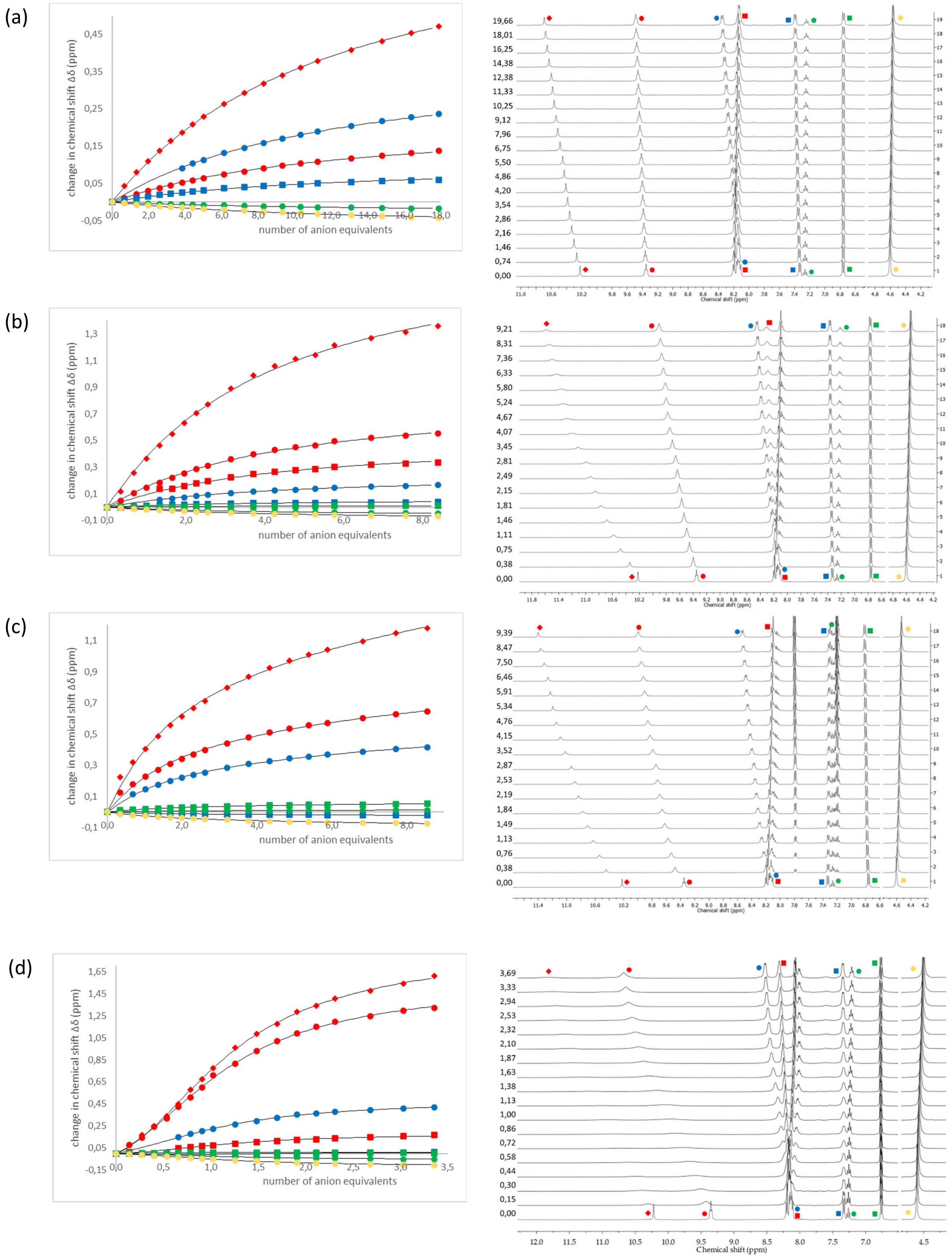

Figure S9. Corresponding experimental chemical shift changes (symbols) and calculated binding isotherms (lines) assuming mixed 1:1 + 1:2 (host:guest) binding model (left); Stacked plots from ^{1}H NMR titrations of receptor *trans*-**1** with increasing amount of **(a)** TBACl, **(b)** $TBAMeCO_2$, **(c)** $TBAPhCO_2$, and **(d)** $TBAH_2PO_4$ in DMSO-d_6 + 0.5% H_2O (v/v) (right).

(a)

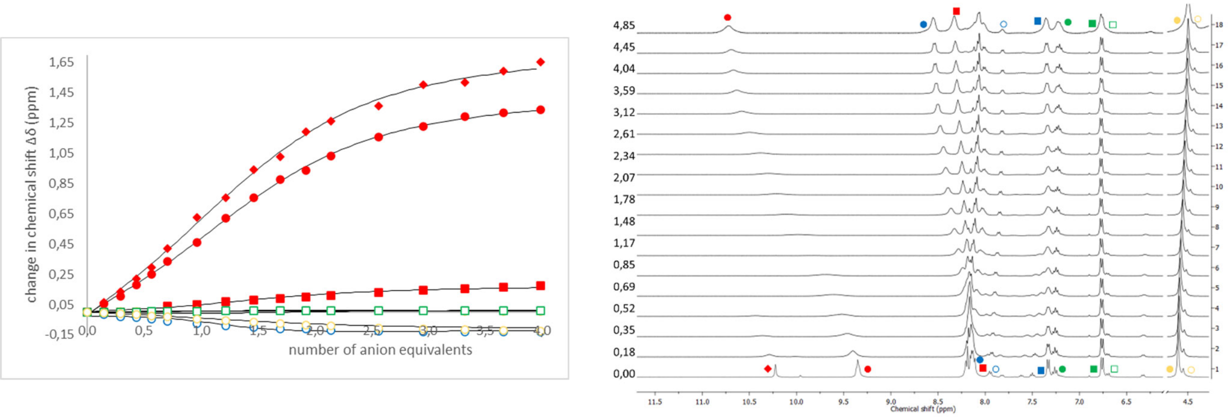

Figure S10. Corresponding experimental chemical shift changes (symbols: closed for present *trans*-**1**, open for *cis*-**1**) and calculated binding isotherms (lines) assuming 1:1 binding model for *cis*-**1** and mixed 1:1 + 1:2 binding model for *trans*-**1** (left); Stacked plots from ^{1}H NMR titrations of receptor *cis*-**1** with increasing amount of $TBAH_2PO_4$ in DMSO-d_6 + 0.5% H_2O (v/v) (right).

3. X-ray measurements details

<u>Experimental details for the crystal *trans*-**1**·$(H_2O)_2$·MeOH</u>:

The X-ray measurement of *trans*-**1** was performed at 100(2) K on a Bruker D8 Venture Photon100 diffractometer equipped with a TRIUMPH monochromator and a MoKα fine focus sealed tube (λ = 0.71073 Å). A total of 1740 frames were collected with Bruker APEX2 program[4] and the frames were integrated with the Bruker SAINT[5] software package using a narrow-frame algorithm. Integration of the data using a monoclinic unit cell yielded a total of 55282 reflections to a maximum ϑ angle of 25.05° (0.84 Å resolution), of which 5919 were independent (average redundancy 9.340, completeness = 99.8%, R_{int} = 3.85%, R_{sig} = 2.29%) and 4637 (78.34%) were greater than 2σ(F^2). The final cell constants of a = 11.9554(8) Å, b = 10.5001(7) Å, c = 26.6855(17) Å, β = 91.1152(17)°, V = 3349.3(4) Å^3, are based upon the refinement of the XYZ-centroids of 9964 reflections above 20 $\sigma(I)$ with 5.966° < 2ϑ < 50.64°. Data were corrected for absorption effects using the multi-scan method (SADABS).[6] The ratio of minimum to maximum apparent transmission was 0.934. The calculated minimum and maximum transmission coefficients (based on crystal size) are 0.975 and 0.994.

The structure was solved and refined using SHELXTL Software Package[7] using the space group $P2_1/n$, with Z = 2 for the formula unit, $C_{65.05}H_{78.22}N_{14}O_{18.66}$. The final anisotropic full-matrix least-squares refinement on F^2 with 485 variables converged at R1 = 4.36%, for the observed data and wR2 = 11.29% for all data. The goodness-of-fit was 1.020. The largest peak in the final difference electron density synthesis was 0.495 e$^-$/Å^3 and the largest hole was -0.292 e$^-$/Å^3 with an RMS deviation of 0.044 e$^-$/Å^3. On the basis of the final model, the calculated density was 1.343 g/cm^3 and F(000), 1432 e$^-$. Structure beside of pincer-type host *trans*-**1** located at the inversion center and one fully ordered H_2O molecule contains disordered solvent moieties modeled as methanol and water species with refined occupancies. Both

MeOH and H_2O moieties share the same space with refined occupancy ratio yielding 0.53(1):0.47(1). To preserve reasonable geometry of these MeOH and H_2O molecules C-O and O-H distance restraints were used. Two additional water molecules with refined occupancy lower than 30% were left without H atoms assigned. Numerous short O...O contacts result from disorder of the solvent molecules. Due to the disorder of the solvent molecules structure contains non-integer number of atoms.

All ordered and major-component disordered non-H atoms were refined anisotropically. Most of hydrogen atoms were placed in calculated positions and refined within the riding model. The temperature factors of all hydrogen atoms were not refined and were set to be equal to either 1.2 or 1.5 times larger than U_{eq} of the corresponding heavy atom. Positions of hydrogen atoms engaged in hydrogen bonds, were refined.

The atomic scattering factors were taken from the International Tables.[8] Molecular graphics was prepared using program Diamond 3.2.[9] Thermal ellipsoids parameters are presented at 50% probability level.

Experimental details for the crystal *trans*-**1**·$(H_2O)_3$:

The X-ray measurement of *trans*-1 was carried out at 100 K on a SuperNova Agilent diffractometer using CuKα (λ=1.54184 Å) radiation. Data reduction was done with CrysAlisPro (Agilent Technologies, Version 1.171.35.21b). The structures were solved by SUPERFLIP[10] and refined using the SHELXL[11] Software Package.

Crystallographic data for presented crystals have been deposited with the Cambridge Crystallographic Data Centre (CCDC). These data can be obtained, free of charge, from CCDC [e-mail: deposit@ccdc.cam.ac.uk or fax: +44(0)-1223−336033].

Table S2. Crystal data and structure refinement details for *trans-**1***·$(H_2O)_2$·MeOH (solvate a) and *trans*-**1**·$(H_2O)_3$ (solvate b)

Name	*Trans*-**1** solvate a	*Trans*-**1** solvate b
Empirical formula	$C_{65.05}H_{78.22}N_{14}O_{18.66}$	$C_{64}H_{70}N_{14}O_{20}$
Formula weight	1354.84	1355.34
CCDC No.	2128868	2121303
Wavelength	0.71073	1.54184
Crystal system	Monoclinic	Triclinic
Space group	$P2_1/n$	*P*-1
Unit cell dimensions	*a* = 11.9554(8) Å<br>*b* = 10.5001(7) Å<br>*c* = 26.6855(17) Å	*a* = 10.9112(5) Å<br>*b* = 13.2394(6) Å<br>*c* = 14.1760(6) Å
Volume	3349.3(4) Å^3	1894.4(1) Å^3
Z	2	1
Density Calc.	1.343 g/cm^3	1.188 g/cm^3
Mu (mm^{-1})	0.100	0.755
F(000)	1432	712
Crystal	orange prism	orange plate
Crystal size	0.26 × 0.19 × 0.06 mm^3	0.2 × 0.1 × 0.02 mm^3
Reflections collected (all / independent)	55282/5919	12375/7075
Absorption correction	multi-scan	multi-scan
Refinement method	Full-matrix least-squares on F^2	Full-matrix least-squares on F^2
Parameters	485	472
Goodness-of-fit on F^2	1.020	1.089

a)

b)

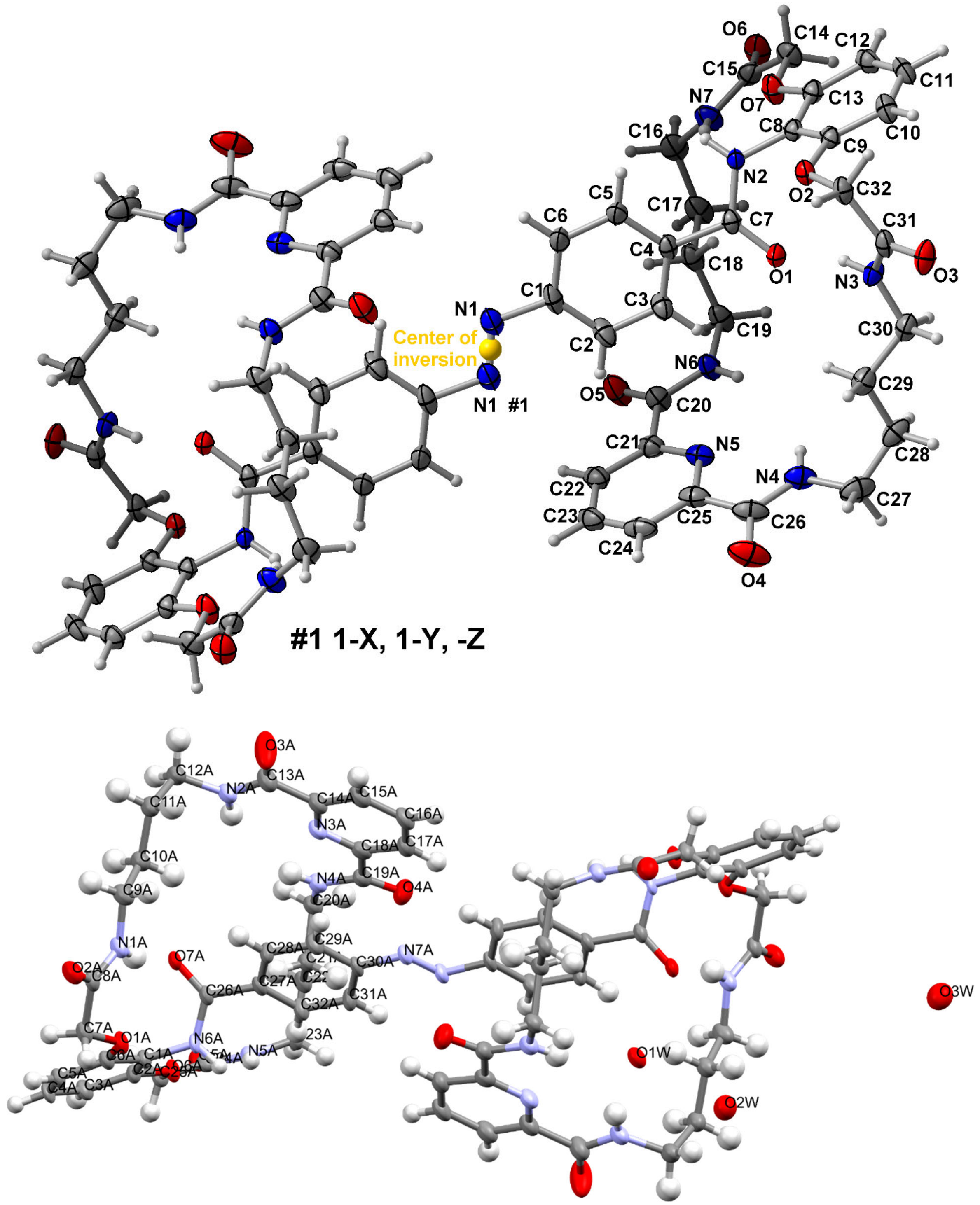

Figure S11. Thermal ellipsoid plot (ORTEP) diagrams for crystals *trans*-**1**·$(H_2O)_2$·MeOH (a) and *trans*-**1**·$(H_2O)_3$ (b); thermal ellipsoids shown at a 50% probability level.

4. References

1. Rodríguez-Barrientos, D.; Rojas-Hernández, A.; Gutiérrez, A.; Moya-Hernández, R.; Gómez-Balderas, R.; Ramírez-Silva, M.T. Determination of pKa values of tenoxicam from 1H NMR chemical shifts and of oxicams from electrophoretic mobilities (CZE) with the aid of programs SQUAD and HYPNMR. *Talanta* **2009**, *80*, 754-762, doi:10.1016/j.talanta.2009.07.058.
2. Lowe, A.J.; Pfeffer, F.M.; Thordarson, P. Determining binding constants from 1H NMR titration data using global and local methods: a case study using [n]polynorbornane-based anion hosts. *Supramol. Chem.* **2012**, *24*, 585-594, doi:10.1080/10610278.2012.688972.
3. Thordarson, P. Determining association constants from titration experiments in supramolecular chemistry. *Chem. Soc. Rev.* **2011**, *40*, 1305-1323, doi:10.1039/C0CS00062K.
4. APEX2,. Bruker AXS Inc., Madison, Wisconsin, USA, 2013.
5. SAINT,. Bruker AXS Inc., Madison, Wisconsin, USA, 2013.
6. SADABS,. Bruker AXS Inc., Madison, Wisconsin, USA, 2012.
7. G. M. Sheldrick, Acta Crystallogr. 1990, A46, 467-473; Sheldrick, G. M. Acta Cryst., 2008, A64, 112–122.
8. Wilson, A.J.C.; Geist, V. International Tables for Crystallography. Volume C: Mathematical, Physical and Chemical Tables. Kluwer Academic Publishers, Dordrecht/Boston/London 1992 (published for the International Union of Crystallography), 883 Seiten, ISBN 0-792-3-16-38X. *Cryst. Res. Technol.* **1993**, *28*, 110-110, doi:10.1002/crat.2170280117.
9. Diamond – Crystal and Molecular Structure Visualization Crystal Impact – K. Brandenburg & H. Putz GbR, Rathausgasse 30, D-53111 Bonn, 2012.
10. Palatinus, L.; Chapuis, G. SUPERFLIP − a computer program for the solution of crystal structures by charge flipping in arbitrary dimensions. J. Appl. Crystallogr. 2007, 40, 786−790.
11. Sheldrick, G. M. Crystal structure refinement with SHELXL. Acta Crystallogr., Sect. C: Struct. Chem. 2015, C71, 3−8.
